# Ultraviolet Sensing-Guided Biomedical Systems: From Label-Free Imaging to Dosimetry and Therapy Feedback

**DOI:** 10.3390/bios16060322

**Published:** 2026-06-02

**Authors:** Haosong Du, Yunxin Wang, Ruochong Zhang, Malini Olivo, Renzhe Bi

**Affiliations:** 1 Department of Biomedical Engineering, College of Design and Engineering, National University of Singapore, Singapore 119077, Singapore; e1582992@u.nus.edu; 2 Agency for Science, Technology and Research (A*STAR) Skin Research Labs, 31 Biopolis Way, #07-01 Nanos, Singapore 138669, Singapore; zhang_ruochong@a-star.edu.sg; 3 College of Medicine and Biological Information Engineering, Northeastern University, Shenyang 110169, China; wangyx52@mails.neu.edu.cn

**Keywords:** ultraviolet (UV), ultraviolet sensing, biosensors, UV imaging, UV phototherapy, UV dosimetry, Far-UVC, UV-PAM, MUSE, wearable sensors, AI-assisted diagnosis

## Abstract

Ultraviolet (UV) light is emerging as an important tool for biosensing, biomedical signal readout, and dose monitoring because of its strong and selective interactions with nucleic acids, proteins, and other biological components. This review summarizes recent progress in UV sensing-guided biomedical systems, with emphasis on three interconnected directions: label-free and surface-weighted imaging, wearable and embedded UV dosimetry, and sensor-assisted therapeutic guidance. Representative examples include ultraviolet photoacoustic microscopy (UV-PAM) for label-free nuclear imaging, microscopy with ultraviolet surface excitation (MUSE) for rapid slide-free histology-like readout, epidermal and flexible UV dosimeters for skin-level exposure quantification, and UV therapeutic platforms that are increasingly supported by sensing, dosimetry, and feedback for safer dose delivery. Across these applications, we emphasize the shared biosensing principles of signal generation, optical or acoustic transduction, quantitative readout, calibration, and feedback-informed decision support. We also discuss the role of artificial intelligence in virtual staining, image enhancement, domain correction, dose prediction, and decision support. The review concludes with key translational challenges in standardization, uncertainty quantification, multimodal integration, and feedback-driven system design. Overall, this sensing-centered perspective helps define the role of UV technologies more clearly within biosensors-oriented biomedical engineering.

## 1. Introduction

Ultraviolet (UV) radiation (approx. 10–400 nm) has long occupied a unique and central position in biomedical engineering, stemming from its profound interactions with biological macromolecules and the consequent significant biological effects. Compared with visible and near-infrared (NIR) light, UV light provides stronger intrinsic biochemical contrast, shallower excitation confinement, and greater sensitivity to dose-dependent biological responses. Depending on wavelength, irradiance, pulse duration, and target properties, UV irradiation may also engage different physical mechanisms, including electronic transitions, thermal processes, and non-linear absorption-related responses [1]. These properties make UV particularly suitable for applications that require label-free molecular contrast, surface-weighted readout, and direct dose quantification at the tissue interface. Historically, UV applications have predominantly bifurcated into two domains. The first is phototherapy and dermatology: from Niels Ryberg Finsen’s Nobel Prize-winning work in 1903 utilizing concentrated light for the treatment of lupus vulgaris, to the Goeckerman regimen combining coal tar with broad-spectrum UV for psoriasis; these milestones established the clinical feasibility of UV administration within strict therapeutic windows [2]. The second domain is disinfection and sterilization: typical UVC (commonly 254 nm) is widely employed for the microbial inactivation of air, water, and surfaces due to its strong absorption by nucleic acids; however, its application has always been accompanied by stringent safety constraints regarding potential ocular and cutaneous hazards [3]. Consequently, throughout much of the 20th century, medical UV technology primarily manifested as a bio-effect-oriented, open-loop modality, characterized by a lack of precise spatial control, real-time dosimetric feedback, and deep integration with data-intensive clinical workflows.

This article was developed as a structured narrative review rather than a formal systematic review focused on a single narrowly defined question, consistent with established distinctions among review types and with current expectations for transparent narrative review reporting [4,5]. To improve methodological transparency, literature identification was performed through structured searches of PubMed, Web of Science, and Scopus, supplemented by Google Scholar searches and backward citation screening of relevant reviews and primary studies, while drawing on general reporting principles that emphasize transparent review processes [6]. The search scope focused mainly on publications from 2010 onward, with particular emphasis on work published after 2015, while earlier landmark studies were retained when necessary to establish historical or mechanistic context. Search terms combined “ultraviolet” or “UV” with domain-specific terms including “photoacoustic microscopy”, “UV-PAM”, “microscopy with ultraviolet surface excitation”, “MUSE”, “wearable UV sensor”, “UV dosimetry”, “Far-UVC”, “UV phototherapy”, “calibration”, “artificial intelligence”, and “clinical translation”. Studies were selected on the basis of technical relevance, representativeness, and translational significance across four interconnected domains: UV-PAM, MUSE, wearable and embedded UV dosimetry, and sensing-assisted UV therapeutic systems. Narrative synthesis was then organized around shared dimensions of physical mechanism, transduction, quantitative readout, calibration, AI-assisted interpretation, and translational relevance [5]. In total, approximately 250 records were initially screened across database searches, Google Scholar searches, and backward citation tracking. After removing duplicates and excluding studies outside the scope of UV-enabled biomedical engineering, approximately 100 publications were retained for detailed evaluation and synthesis.

Since approximately 2015, the role of UV in biomedicine has increasingly shifted from simple light delivery toward sensing-enabled and readout-oriented system design, driven by the parallel maturation of several key technology chains. At the source level, major advances in the external quantum efficiency and operational reliability of AlGaN-based deep ultraviolet (DUV) light-emitting diodes (LEDs) have enabled miniaturized, wavelength-selective, and electronically tunable UV illumination, reducing dependence on mercury lamps and making UV sources more compatible with integrated sensing platforms [7,8]. In parallel, progress at the detector and system levels, including high-sensitivity photodetectors, optimized acoustic detection for photoacoustic imaging, and miniaturized electronic architectures, has substantially strengthened UV-related signal acquisition, transduction, and quantitative readout capabilities.

At the materials and device level, the emergence of flexible electronics and wireless, battery-free platforms has further extended UV technology into the domain of wearable and skin-interfaced biosensing, enabling direct and continuous monitoring of UV exposure and therapeutic dose at the skin interface rather than relying solely on environmental measurements or indirect estimation [9]. Meanwhile, rapid developments in computational imaging and artificial intelligence have improved the interpretability of UV-derived signals by transforming shallow-penetration, label-free, and signal-limited data into clinically meaningful representations, such as virtual histology [10]. Recent reviews of ultraviolet microscopy further indicate that progress in UV illumination, multimodal integration, and AI-assisted virtual staining is expanding the practical scope of UV-enabled biomedical imaging [11]. Taken together, these developments have moved UV from a modality used mainly for irradiation toward a biomedical systems framework defined by signal generation, quantitative readout, calibration, and data-guided interpretation.

Against this backdrop, a distinct ecosystem of UV technologies that has rapidly formed and expanded post-2015 constitutes the core of this review. These technologies include (i) ultraviolet photoacoustic microscopy (UV-PAM), which utilizes the strong endogenous absorption of DNA/RNA to achieve label-free, nuclear-resolution imaging [12]; (ii) microscopy with UV Surface Excitation (MUSE), which leverages the extremely shallow penetration depth of UV to rapidly generate histology-like images without the need for slide preparation, naturally adapting to intraoperative and low-resource workflows [13]; (iii) wearable and embedded UV dosimetry, enabling personalized, continuous quantification of environmental or therapeutic UV exposure [9]; and (iv) UV therapeutic and preventive systems that are increasingly coupled with sensing, dosimetry, and computational support, including Far-UVC disinfection and AI-assisted phototherapy [14]. In this review, these therapeutic systems are not treated as biosensors in the strict classical sense. Rather, they are discussed a sensing-assisted or biosensing-adjacent systems in which UV delivery is progressively informed by measurement, calibration, and feedback. Here, “biosensing-adjacent” refers to UV-based therapeutic or preventive platforms that do not perform biosensing as their primary function, but incorporate sensing, dosimetry, calibration, imaging, or computational monitoring to support safer and more controllable biomedical use.

Recent publication trends also indicate sustained growth across these directions. Research on ultraviolet germicidal irradiation (UVGI) has shown continued growth over the past decade and has expanded from early work on inactivation mechanisms and dose-response relationships to topics such as disinfection in occupied spaces, Far-UVC safety assessment, and UVC-LED device engineering [15]. Similarly, the scale and interdisciplinary penetration of photoacoustic imaging research in biomedical engineering have continuously risen, with UV-related directions expanding from proof-of-concept stages to application scenarios with greater translational potential, such as label-free histological imaging, pathological interpretation, and clinical workflow adaptation [16].

Despite the rapid progress in this field, existing reviews remain notably fragmented in their overall perspective. Most works focus on singular themes—such as UV disinfection mechanisms, photoacoustic imaging physics, or specific imaging techniques like MUSE—while rarely discussing imaging, dosimetry, therapy, and artificial intelligence within a unified biomedical engineering framework [3,13,16]. In addition, many existing reviews are organized by application scenario or wavelength range rather than by sensing logic, platform design, and system capability. This limits discussion of cross-cutting issues such as quantitative readout, calibration, safety margins, workflow integration, and feedback-informed decision-making. To address this gap, the present review examines emerging UV technologies through the shared dimensions of physical mechanism, engineering implementation, quantitative readout, calibration, AI-assisted analysis, and translational potential. The main contribution of this review is therefore not only to summarize recent progress in individual UV technologies but also to interpret otherwise heterogeneous platforms within a common biosensing-informed framework. In this framework, the connection to biosensing is strongest for imaging and dosimetry, and more qualified for therapeutic applications, where many systems remain open-loop and only a limited number of feedback-enabled strategies have been demonstrated. To clarify the positioning of the present review relative to prior review literature, Table 1 compares representative existing reviews across scope, calibration, AI integration, and translational emphasis.

## 2. Ultraviolet Photoacoustic Microscopy (UV-PAM)

Ultraviolet photoacoustic microscopy (UV-PAM) is an emerging UV-enabled sensing and imaging modality that integrates optical excitation with ultrasonic detection to achieve label-free, high-resolution imaging of tissue microstructures. By leveraging short-pulsed ultraviolet lasers, most commonly around 266 nm, to excite the strong endogenous absorption of nucleic acids, UV-PAM generates photoacoustic signals from cell nuclei without the need for exogenous contrast agents or conventional histological staining. This endogenous nuclear contrast is the key reason why UV-PAM is relevant to the present review: it allows biologically meaningful microstructural information to be generated, transduced, and quantitatively interpreted within a sensing-oriented framework. The system coordinates focused optical excitation with ultrasonic detection to acquire high-contrast nuclear images while preserving the native physiological state of biological specimens. Accordingly, UV-PAM can be viewed not only as an optical imaging technique but also as a UV-enabled readout platform in which endogenous molecular absorption is converted into interpretable histology-like information. With ongoing advances in system engineering and AI, UV-PAM is moving from laboratory prototyping toward applications in intraoperative diagnosis, rapid pathology, and virtual histology.

### 2.1. Physical Principle

The fundamental capability of UV-PAM relies on the distinct absorption contrast of nucleic acids in the ultraviolet band. At 266 nm, DNA and RNA exhibit substantially higher molar extinction coefficients than most surrounding cytoplasmic proteins, including aromatic amino acid-containing components such as tryptophan and tyrosine. Upon pulsed UV excitation, this differential absorption is converted into localized heat through non-radiative relaxation, which in turn induces thermoelastic expansion and generates acoustic waves detectable by an ultrasonic transducer. This signal-generation pathway enables UV-PAM to visualize cell nuclei directly from endogenous absorption contrast, which is why it can achieve label-free nuclear imaging without the addition of dyes, stains, or molecular probes. As a result, cell nuclei appear as high-contrast structures against the surrounding background, functionally resembling the nuclear selectivity of hematoxylin in standard H&E staining [12,24].

**Figure 1:**
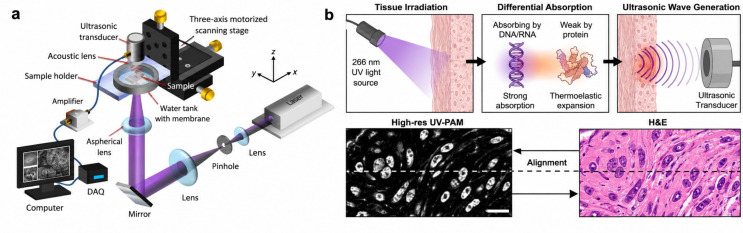
Schematic and principle of UV-PAM. (**a**) Schematic diagram of the UV-PAM experimental setup, showing the optical path, ultrasonic transducer, and scanning stage. (**b**) Principle of label-free histological imaging. The 266 nm UV light is strongly absorbed by DNA/RNA in cell nuclei, inducing thermoelastic expansion and generating ultrasonic waves. The bottom panels demonstrate the validation of UV-PAM against standard H&E staining, showing excellent alignment of cellular structures. (Reprinted from ref. [27]).

Regarding spatial resolution, UV-PAM operates as an optical-resolution technique (OR-PAM), achieving sub-micron lateral resolution (approximately 0.5–1.2 µm) determined by the diffraction limit of the focused UV beam rather than the ultrasonic transducer’s aperture. This enables confocal-like 3D tomography of nuclear morphology and density—critical metrics for pathological assessment—without requiring complex confocal pinholes [25,26]. The combination of strong UV attenuation in tissue and the shallow focal range of high-NA optical excitation also provides intrinsic optical sectioning, allowing UV-PAM to generate depth-resolved nuclear information in a manner that is particularly relevant to histology-like assessment. This capability is important for pathology-oriented applications because it supports the visualization of nuclear morphology, density, and architectural distribution without the need for physical sectioning or complex confocal filtering. The schematic setup and label-free nuclear imaging principle of UV-PAM are illustrated in Figure 1.

### 2.2. Engineering-Driven Evolution and System Innovation

Although UV-PAM inherently possesses physical advantages such as nucleic acid-specific absorption, high lateral resolution, and optical-sectioning capabilities, its early implementation was primarily confined to proof-of-concept stages in laboratory settings, remaining insufficient for practical biomedical research and clinical applications. The critical impediments to the practicalization of UV-PAM were not its fundamental physical principles, but rather a series of challenges at the system engineering level, including limited imaging depth, restricted imaging speed, insufficient field of view (FOV) and depth of field (DOF), and incompatibility with clinical workflows. Over the past decade, the technological evolution of UV-PAM has clearly demonstrated an engineering-driven trajectory: significant advancements have originated from responses to specific application demands rather than mere optimization of physical parameters. This section systematically outlines the key developmental stages of UV-PAM from concept verification to clinically relevant imaging technologies, centering on these core engineering bottlenecks. Table 2 and Table 3 summarize this progression, showing how UV-PAM advanced from submicron nuclear imaging validation to clinically relevant, slide-free throughput through successive engineering optimizations in speed, contrast, and robustness.

#### 2.2.1. Early Validation

The engineering evolution of UV-PAM began with the validation of its capability for nuclear imaging. Yao et al. first proposed utilizing the ultraviolet band to excite the endogenous absorption of DNA and RNA, achieving label-free photoacoustic microscopy of cell nuclei [12]. This work established the feasibility of UV-PAM as a potential virtual histology modality at the conceptual level, demonstrating the acquisition of nuclear morphological information highly relevant to pathological diagnosis without the need for staining.

Subsequently, Yao et al. systematically investigated the effects of UV excitation wavelength on nuclear contrast, signal-to-noise ratio (SNR), and safety dosage, elucidating the optimal trade-off relationship within the ~250–266 nm band for in vivo nuclear imaging [24]. This research provided a clear parametric basis for the engineering implementation of UV-PAM, transitioning it from simple concept verification to a reproducible and controllable imaging technology. Meanwhile, a systematic summary of the photoacoustic microscopy framework [26] further clarified the technical positioning of UV-PAM within the optical-resolution photoacoustic microscopy (OR-PAM) ecosystem.

#### 2.2.2. System Architecture

Early UV-PAM systems relied primarily on transmission-mode illumination geometries. While this configuration established nucleus-specific imaging through endogenous DNA/RNA contrast, its strict constraints on sample thickness hindered applications in thick in vivo tissues or intraoperative specimens. Consequently, shifting the illumination and detection configuration toward reflection-mode architectures became a critical path for clinical translation. Although reflection-mode PAM had established a general paradigm for coaxial opto-acoustic alignment, implementing a reliable UV light-delivery path introduced unique material-physics challenges: standard optical glasses (e.g., BK7) exhibit strong UV absorption, and conventional optical adhesives are prone to photodamage or background fluorescence under 266 nm excitation. Addressing this, Wong et al. established the practical foundation for reflection-mode UV-PAM by replacing traditional lenses with all-fused-silica components and adopting an air-gap assembly strategy to eliminate adhesive interference, thereby mitigating UV-specific optical loss and damage and stabilizing UV illumination delivery [27].

However, as resolution demands increased, conventional reflection-mode illumination schemes encountered structural bottlenecks. Existing opto-acoustic beam combiners or ring-transducer-based light delivery often restricted the usable optical numerical aperture (NA), resulting in an intractable trade-off among lateral resolution, detection sensitivity, and system compactness. Recently, Kim et al. achieved a breakthrough in axial illumination delivery by developing a UV-transparent ultrasound transducer (UV-TUT). This configuration enables UV excitation light to transmit directly through the transducer, allowing high-NA optical focusing in a highly compact footprint. By reconfiguring the illumination pathway, this design effectively relaxes the resolution–sensitivity trade-off, improves the resolving power for nuclear structures, and provides a more optimized interface for future multimodal integration compatible with visible and near-infrared bands [28].

#### 2.2.3. Throughput Scaling

Imaging throughput represents a critical bottleneck restricting the transition of UV-PAM from laboratory prototypes to clinical pathology applications. Traditional point-scanning UV-PAM, limited by the inertia of mechanical scanning components and the inherent dead time of the “step-and-shoot” mode, resulted in excessive time consumption for large-field imaging (millimeter scale and above), failing to meet the timeliness requirements for rapid intraoperative assessment or large-scale tissue screening.

To address this efficiency pain point at the system level, Baik et al. incorporated waterproof micro-electromechanical systems (MEMS) scanning technology, enabling rapid data acquisition over large fields of view through high-speed scanning and thereby markedly improving the practicality of reflection-mode UV-PAM for intraoperative pathological workflows [29]. Building on further optimization of the temporal dimension, Li et al. proposed a high-speed UV-PAM architecture based on continuous scanning, effectively eliminating the dead time of traditional stepping by reconstructing the synchronization mechanism between scanning trajectories and laser triggering. This method significantly improved the data acquisition duty cycle while maintaining high nuclear contrast and sub-micron resolution, achieving efficient histology-like imaging of unprocessed biological tissues [30]. Furthermore, to break through the physical speed limits of single-point mechanical scanning, Cao et al. introduced a parallelization strategy in the spatial dimension [31]. This work proposed a Parallel UV-PAM scheme, utilizing a multifocal array to achieve parallel excitation and detection. This “space-for-time” design philosophy increased imaging throughput to near-clinically acceptable levels without significantly increasing system complexity. This technological leap from single-point serial to multi-point parallel scanning marks the progressive evolution of UV-PAM into a scalable high-throughput histopathology scanner, equipping it with detection capabilities covering the whole-slide scale [31].

#### 2.2.4. Specimen Robustness

In real-world clinical pathology scenarios, fresh tissue samples frequently exhibit irregular surface topologies and non-uniform thickness distributions, posing severe challenges to the imaging stability of UV-PAM. The necessity of employing high numerical aperture (NA) objectives to ensure sub-cellular lateral resolution inevitably results in an extremely shallow DOF. This inherent physical contradiction between resolution and DOF renders imaging quality highly sensitive to sample flatness, leading to susceptibility to defocus artifacts and compromising diagnostic consistency.

To reconcile this physical limitation and enhance system robustness, researchers have introduced hybrid strategies synergizing computational optics with advanced nanophononics. At the system and algorithmic level, He et al. proposed an extended depth-of-field (EDOF) imaging scheme that, through the interplay of hardware design and computational reconstruction, significantly expands the axial focusing range without sacrificing resolution [32]. Concurrently, hardware innovations focusing on wavefront engineering have emerged as a pivotal direction. Zhao et al. leveraged ultrathin metalenses based on metasurface technology to achieve extended DOF within a highly compact footprint through sub-wavelength phase modulation [33]. Similarly, Chen and Song utilized liquid crystal diffractive optical elements to precisely shape the optical wavefront, successfully extending the DOF to the scale of hundreds of micrometers and effectively decoupling the constraints between lateral resolution and axial depth [34]. Furthermore, to address the strong scattering attenuation of UV light in thick tissues, Li et al. demonstrated the utility of tissue clearing as a critical auxiliary method; by homogenizing the tissue refractive index, this approach reduces photon scattering paths in turbid media, thereby enhancing contrast in deep-layer imaging [25]. Collectively, these DOF extension schemes—which enhance robustness against morphological variations—complement parallel scanning strategies that optimize temporal efficiency, together expanding the operational boundaries of UV-PAM for intraoperative non-destructive pathology [31,33,34].

**Table 2:** Engineering evolution and representative performance characteristics of UV-PAM.

Ref.	Year	Geometry	UV (nm)	Key System Innovation	Representative Performance *	Specimen	AI Layer
[12]	2010	Transmission	266	DNA/RNA endogenous contrast demonstrated	Lateral: sub-µm (reported)	Cells/thin tissue	No
[24]	2012	Transmission	245–275 (opt.)	Optimal UV band for nuclear contrast/safety	Optimal λ reported	In vivo mouse	No
[27]	2017	Reflection	266	UV-compatible reflection-mode design (UV optics/material strategy)	Histology-like multilayer imaging (reported)	Human breast (fresh)	No
[30]	2020	Reflection	266	Continuous scanning/reduced step-and-shoot dead time	Throughput improved (reported)	Fresh tissue	No
[29]	2021	Reflection	266	Waterproof MEMS fast scanning (workflow-oriented)	Near real-time ROI imaging (reported)	Clinical specimens	No/optional colorization
[35]	2022	Reflection	266	Thick/irregular specimen handling + clinical workflow	Clinical task-driven performance (reported)	Bone/clinical	Yes (virtual staining/DL assist)
[25,36]	2022 (a/b)	Reflection	266	(a) Tissue clearing for contrast (b) Virtual staining pipeline	CNR gain OR H&E-like output (reported)	Cleared/fresh	(a) No (b) Yes
[28]	2024	Reflection	266	UV-transparent ultrasound transducer (UV-TUT)	Higher NA/improved resolution (reported)	Thick fresh tissue	Optional
[31]	2024	Parallel (multifocal)	266	Multi-foci parallelization + extended DOF beam strategy	Speed: 0.4 mm^2^/s; Lateral: 1.3 µm	Slide-free fresh tissue	No
[37]	2025	Reflection	266	High-NA optics + fine scanning	Lateral: 240 nm; AUC ~0.90	Human liver	Yes (GAN + CNN/CAD)
[32]	2025	Reflection	266	EDOF via computational optics + self-calibration	Effective DOF expanded (reported)	Uneven tissue	Yes (computational)
[33]	2023	(Optical module)	UV	UV metalens elongated DOF	DOF extended (reported)	—	—
[34]	2025	Reflection	UV	LC diffractive wavefront shaping	DOF to 100s µm (reported)	Thick/uneven	Optional

Note: * Values in the “Representative Performance” column are presented as representative reported capabilities under study-specific conditions and should not be interpreted as directly normalized head-to-head comparisons across platforms, laboratories, or specimen types.

**Table 3:** Quantitative milestones of UV-PAM evolution.

Engineering Bottleneck	Ref.	Key Innovation (System)	Performance Metric	Quantitative Value	Compared to	Key Impact
Unverified feasibility of label-free nuclear imaging	[12]	Endogenous UV excitation at 266 nm (UV-PAM)	Lateral resolution (FWHM)	0.70 ± 0.04 µm	Bead calibration	Established feasibility of label-free nuclear imaging
			Axial resolution (FWHM)	28.5 ± 0.8 µm	System axial response	Baseline depth-sectioning capability
			Pulse energy (behind membrane)	35 nJ	System measurement	Energy reference for safety analysis
			Minimum scan step	0.31 µm	Scanner limit	Defines subcellular sampling density
Need parametric basis for in vivo nuclear imaging	[24]	Wavelength optimization	Optimal UV wavelength	250 nm	245–275 nm sweep	Defines optimal excitation band
			Minimum pulse energy	2 nJ	Low-energy operation	Guides safety–contrast trade-off
Transmission geometry; limited throughput	[27]	Reflection-mode UV-PAM on human tumor	Raster scan time	~100 min (5 × 5 mm^2^)	Same-system baseline	Quantifies throughput bottleneck
			ROI scan time	~180 min (10 × 4.2 mm^2^)	Clinical workflow	Demonstrates slide-free feasibility
			Image–histology agreement	Corr. 0.74/0.64	Matched H&E	Quantitative Histology validation
Mechanical inertia; scan dead time	[30]	High-speed GM-UV-PAM	Acquisition time	<15 min (5 × 5 mm^2^, 3 mm thick)	Prior UV-PAM	Brings biopsy-scale imaging into clinical window
			Large-FOV time	~56 min (12 × 8 mm^2^)	Same system	Quantifies scaling cost
			CNR/thickness	49 (3 mm); 5.6 mm @CNR > 10	CNR model	Defines depth–quality envelope
Clinical workflow timeliness	[29]	MEMS-based intraoperative UV-PAM	Wide-field imaging time	240 s (10 × 10 mm^2^)	Step-scan UV-PAM	Enables intraoperative use
			Lateral resolution	~1.2 µm	Optical focus	Maintains cellular detail
Scattering-limited contrast	[25]	Tissue clearing (CUBIC)	Absolute CNR	41.4/35.3/26.0 @6.25 nJ	Uncleared tissue	Enhances deep-layer contrast
			Energy-normalized CNR gain	4.1×/6.7×/3.4×	No clearing	Quantifies clearing benefit
Workflow constraints; shallow DOF	[31]	Parallel UV-PAM (8 foci)	Imaging speed	0.4 mm^2^ s^−1^	Single-focus UV-PAM	Clinically meaningful throughput
			Depth-of-field	40 → 200 µm	Conventional DOF	Robust imaging on uneven tissue
Resolution–sensitivity trade-off	[28]	UV-transparent ultrasound transducer	Lateral resolution	0.47 ± 0.03 µm	Ring-transducer design	Improves nuclear resolving power
Large-DOF wavefront engineering	[33]	UV metalens	Depth of field	220 µm	Conventional objective	Extends in-focus range
	[34]	LC diffractive optics	Depth of field	~250 µm	Conventional DOF	Decouples DOF–resolution trade-off

Note: The quantitative values listed here are intended to illustrate representative engineering bottlenecks and reported milestones in UV-PAM evolution. Because measurement conditions, specimen types, optical geometries, and evaluation protocols differ across studies, these values should not be interpreted as directly normalized cross-study comparisons.

### 2.3. AI-Enhanced UV-PAM

While hardware advancements have significantly bolstered the performance of UV-PAM, physical limits remain regarding imaging speed, image style compatibility, and image quality in low signal-to-noise ratio (SNR) environments. In recent years, the integration of artificial intelligence (AI), particularly deep learning (DL), has introduced a new paradigm of software-defined imaging to UV-PAM. This shift not only resolves challenges that are intractable for hardware alone but also facilitates a transition in the diagnostic dimension from mere imaging to comprehensive analysis.

#### 2.3.1. Virtual Staining

While UV-PAM excels at providing high-contrast nuclear structural information via endogenous absorption, its raw output consists of grayscale maps that differ starkly from the color-coded semantics of standard hematoxylin and eosin (H&E) staining—characterized by purple nuclei and pink cytoplasm—upon which pathologists rely. This visual discrepancy creates a significant cognitive barrier for clinical interpretation, necessitating “virtual staining” as a critical bridge between physical imaging and standard pathological diagnosis [36].

**Figure 2:**
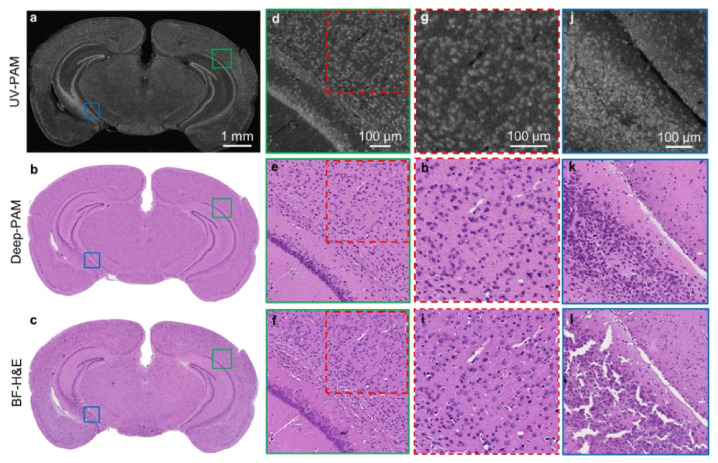
Validation of UV-PAM and Deep-PAM using a mouse brain section. (**a**,**b**) UV-PAM image and its corresponding Deep-PAM image of a 7 μm-thick whole mouse brain section. (**c**) Corresponding BF-H&E image of the same section served as the ground truth for comparison. (**d**–**f**) Zoomed-in images of the green regions in (**a**–**c**). (**g**–**i**) Zoomed-in images of the red dashed regions in (**d**–**f**), used for cellularity analysis. (**j**–**l**) Zoomed-in images of the blue regions in (**a**–**c**). Note the crumbling artifacts in (**l**) caused by xylene treatment during clearing, which are absent in the virtual staining results. (Reprinted from ref. [10]).

Addressing this challenge, Kang et al. introduced Deep-PAM, a deep learning framework utilizing generative adversarial networks (GANs) to achieve real-time style transfer from the photoacoustic grayscale domain to the H&E colour domain [10]. A pivotal engineering breakthrough of this method involves the deployment of Cycle-consistent adversarial networks (CycleGAN) to circumvent the dependency on strictly registered paired data, which are physically infeasible to obtain due to tissue deformation during sectioning. As illustrated in Figure 2, validation using mouse brain sections demonstrates the framework’s capability to transform raw grayscale UV-PAM data (a) into virtually stained H&E images (b). When compared with the standard brightfield H&E reference (c), the virtual staining maintains excellent morphological consistency in cellular density and nuclear distribution. Crucially, it avoids the local crumbling and folding artifacts inherent to physical staining processes. This high-fidelity translation effectively converts the “physical nuclear contrast” of UV-PAM into familiar “histological visual semantics,” thereby enabling pathologists to perform diagnoses without additional training. Furthermore, similar deep learning-based virtual staining strategies have been extended to complex specimens, such as bone tissues, confirming the method’s versatility and robustness for intraoperative rapid pathological assessment [35].

#### 2.3.2. Quality Reconstruction

In UV-PAM, imaging speed often represents a primary bottleneck limiting clinical scalability. This constraint arises not only from the Nyquist sampling requirements intrinsic to point-scanning photoacoustic microscopy but also from stringent limitations imposed by ultraviolet excitation, including restricted pulse repetition rates, limited average power, and photobiological safety considerations. Unlike visible or near-infrared PAM systems that primarily target vascular or functional contrast, UV-PAM focuses on cellular nuclei and subcellular structures, requiring submicrometric lateral resolution and consequently much denser spatial sampling. In large-area, intraoperative imaging scenarios, this combination of high spatial resolution, dense sampling requirements, and limited ultraviolet source performance renders hardware-based acceleration alone insufficient.

Under these UV-PAM-specific constraints, deep learning-based under-sampled reconstruction emerges not as an auxiliary enhancement, but as a critical computational strategy to mitigate structural speed limitations. By intentionally relaxing spatial sampling density during acquisition and compensating for missing high-frequency information during reconstruction, learning-based models exploit prior knowledge of tissue morphology to preserve nuclear continuity and histological interpretability while substantially reducing acquisition time. Previous studies have demonstrated that deep neural networks can recover near-fully sampled photoacoustic microscopy images from highly under sampled data, providing an effective software-level solution to the conventional trade-off between imaging speed and spatial resolution [38].

Beyond individual system implementations, unified reconstruction frameworks such as UPAMNet incorporate stronger physical priors and multiscale feature modelling to improve robustness across different PAM architectures and sampling regimes [39]. This capability is particularly critical for UV-PAM, where the objective is not merely visual image recovery but the preservation of nuclear boundary integrity, tissue texture statistics, and pathological interpretability under sampled conditions. In this context, AI-driven reconstruction effectively transfers the cost of acceleration from hardware to computation, enabling large-area, rapid, and histologically consistent UV-PAM imaging under constrained ultraviolet illumination.

From a review perspective, this paradigm provides clear guidance for future UV-PAM development. Performance gains should not rely exclusively on higher-PRR ultraviolet sources or faster mechanical scanning but instead on coordinated optimization of sampling strategies, system design, and learning-based reconstruction. Such an integrated framework has the potential to redefine the achievable boundaries of speed, resolution, and diagnostic fidelity in UV-PAM, particularly for intraoperative margin assessment and slide-free histological imaging.

#### 2.3.3. Aided Diagnosis

The vision for AI applications in UV-PAM is evolving from singular image quality enhancement toward advanced computer-aided diagnosis (CAD). Based on high-fidelity virtual H&E images, deep learning models are endowed with the capability to automatically recognize pathological features, aiming to mitigate subjective biases arising from fatigue or experiential differences among pathologists via algorithmic objectivity.

**Figure 3:**
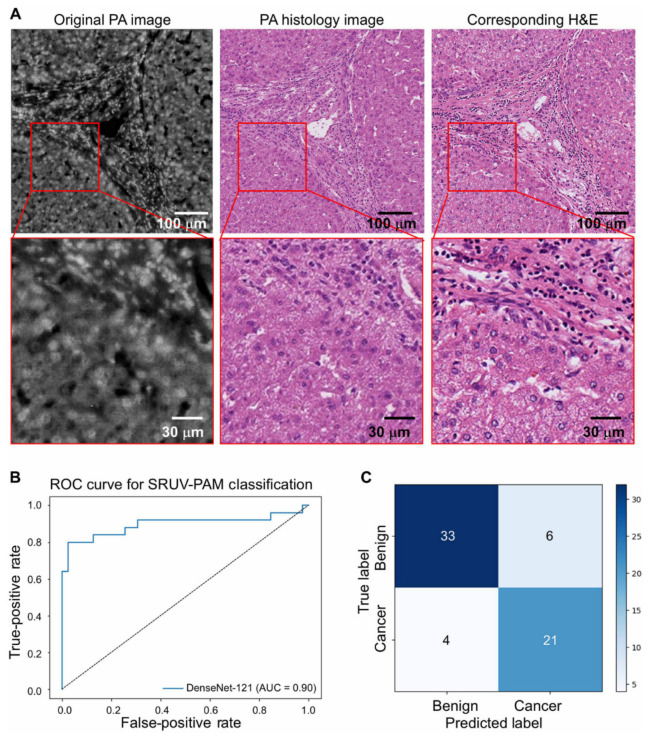
Performance evaluation of deep learning-powered UV-PAM virtual histology and computer-aided diagnosis. (**A**) Validation of high-fidelity virtual pathology: left column shows original grayscale UV-PAM images; middle column shows virtual H&E images generated via deep learning; right column shows corresponding ground-truth H&E-stained sections. The virtual staining accurately reconstructs nuclear and microstructural features of liver tissue, providing a standardized data foundation for downstream AI analysis. (**B**,**C**) End-to-end malignancy classification performance: (**B**) receiver operating characteristic (ROC) curve for automated liver cancer classification using the DenseNet-121 convolutional neural network, achieving an AUC of 0.90, which validates the objectivity of this diagnostic paradigm; (**C**) corresponding confusion matrix demonstrating high accuracy in distinguishing benign from malignant (Cancer) samples. These results strongly support the clinical potential of UV-PAM for intraoperative binary determination of margin status. (Reprinted from ref. [37]).

Recent studies have preliminarily validated the feasibility of this end-to-end diagnostic paradigm. For instance, Park et al. (2025) demonstrated the use of deep convolutional neural networks (e.g., DenseNet-121) to directly classify benign and malignant tissues from UV-PAM images, combining semantic segmentation networks to automatically delineate tumor infiltration boundaries, thereby achieving a binary determination of “margin positive/negative” for intraoperative scenarios [37]. As shown in Figure 3, the end-to-end diagnostic workflow based on UV-PAM virtual H&E not only maintains morphological consistency with real H&E but also realizes malignancy classification and margin assessment through deep networks. Its classification performance reached an AUC of ≈0.90 in the validation set, intuitively reflecting the feasibility and clinical potential of this CAD paradigm. This type of workflow emphasizes an integrated design from data acquisition and model inference to clinical visual feedback, offering more direct clinical decision-making value than simple image display and promising to assist surgeons in achieving precise R0 resection.

### 2.4. Challenges and Future

Translation of UV-PAM remains limited by imaging depth constraints, UV safety considerations, and the difficulty of maintaining quantitative consistency outside controlled laboratory conditions. Strong UV absorption and scattering restrict penetration depth and increase sensitivity to photodamage, which makes it challenging to extend UV-PAM beyond superficial or ex vivo applications. Reflection-mode and coaxial designs improve practicality, but UV-specific optical and packaging issues, including material absorption, parasitic fluorescence, optical loss, and long-term instability of the light-delivery path, continue to affect system reliability.

A further challenge is that UV-PAM is often presented as a high-resolution imaging modality, while its translational value also depends on whether it can provide stable and traceable quantitative readout. In practice, reported performance metrics such as speed, field of view, depth of field, and contrast are often obtained under favorable conditions, whereas robustness to tissue heterogeneity, uneven surfaces, motion, and workflow variability is less consistently demonstrated. For this reason, calibration in UV-PAM should not be treated as an abstract design principle, but as a concrete metrological problem involving optical fluence distribution, acoustic transducer response, acoustic coupling stability, and the reproducibility of absorption-based contrast across devices and specimens.

AI-assisted virtual staining, reconstruction, and diagnosis further improve interpretability and throughput, but they also introduce new constraints related to training-data dependence, domain shift, and failure under specimen or system variation. In particular, virtual staining models must be evaluated not only for visual plausibility but also for preservation of diagnostically relevant features and for the risk of hallucinated or distorted morphology. Future studies should therefore emphasize traceable calibration, cross-device reproducibility, uncertainty-aware AI evaluation, and systematic comparison against histological reference standards. Multi-center validation will be important if UV-PAM is to progress from technically impressive laboratory performance to clinically credible pathology support. Recent multimodal work integrating UV-PAM with spectroscopic autofluorescence further suggests that future translation may depend on combining endogenous absorption contrast with complementary molecular readouts rather than relying on a single signal channel alone [40].

## 3. Microscopy with Ultraviolet Surface Excitation (MUSE)

**Figure 4:**
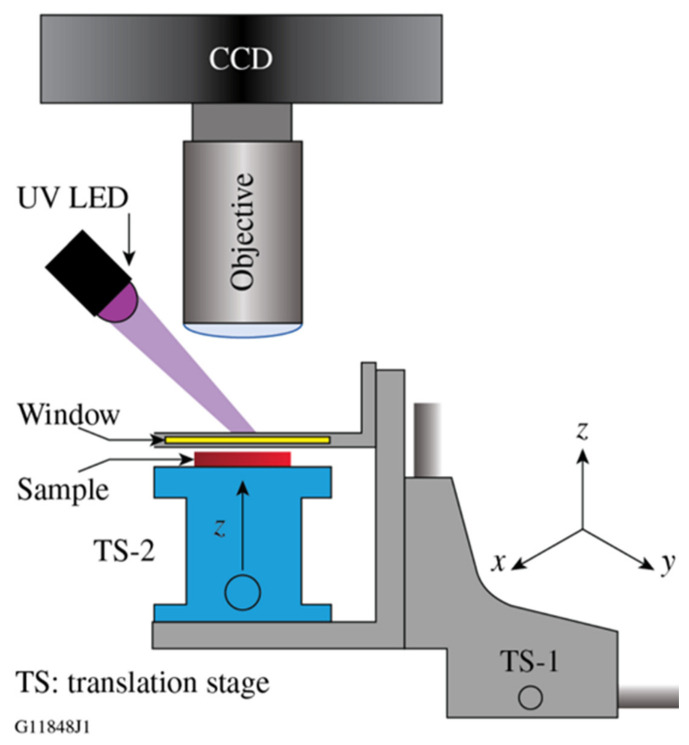
The structure diagram of MUSE. (Reprinted from ref. [41]).

Apart from UV-PAM, microscopy with ultraviolet surface excitation (MUSE) is another surface-weighted optical sensing platform that takes advantage of the shallow penetration of short-wavelength UV light. Using surface-weighted excitation to provide an optical sectioning effect without physical slicing and combining it with rapid surface staining and multi-channel fluorescence contrast, MUSE can produce histology-like images with H&E-like appearance within minutes. Its relevance to the present review lies in the fact that it converts surface-confined UV excitation into interpretable pathological readout through a workflow that is rapid, slide-free, and in many implementations, minimally preparation-dependent. This technology emphasizes a workflow-driven engineering philosophy, making it particularly well suited for intraoperative rapid assessment, margin screening, and point-of-care pathology in low-resource settings [13,17]. Importantly, however, MUSE should be described more precisely as a slide-free and surface-weighted imaging platform than as a uniformly label-free modality, because many practical implementations rely on rapid surface staining to enhance histological contrast. This distinction is important for interpreting both the strengths and the translational constraints of the technique. The schematic diagram is shown in Figure 4.

### 3.1. Physical Principle

The fundamental advantage of MUSE derives from its UV surface excitation–visible fluorescence readout mechanism. Unlike conventional wide-field fluorescence microscopy, in which visible excitation penetrates more deeply and can generate substantial defocus background, MUSE exploits the very high absorption of biological tissue in the short-wavelength UV range, typically below 300 nm. As a result, the effective excitation volume is naturally confined to the superficial tissue layer, usually within only a few to tens of micrometers. This shallow excitation depth is the physical basis for the slide-free character of MUSE, because it enables histology-like surface imaging directly from bulk tissue without conventional sectioning.

In this sense, MUSE achieves a form of optical sectioning through excitation confinement rather than through mechanical slicing or confocal rejection. The use of visible fluorescence readout then allows the superficial morphological and chemical features of the specimen to be captured in a format compatible with pathology-like interpretation.

#### 3.1.1. Spatial Constraint

The imaging foundation of MUSE is built upon the superficial nature of the interaction between UV light and biological tissue. As discussed in Section 2, biological tissues exhibit extremely strong endogenous absorption of short-wavelength UV light. In MUSE technology, this physical characteristic is ingeniously transformed into an advantage: the exceptionally high absorption coefficient restricts the mean free path of UV light in tissue to within 10–20 μm. This rapid attenuation of energy naturally forms an intrinsic optical sectioning effect, allowing MUSE to achieve surface-weighted imaging without relying on confocal pinholes [13]. Unlike confocal microscopy, which filters out defocus light via spatial pinholes to achieve axial resolution, the sectioning capability of MUSE stems directly from the penetration limit of the excitation light: deep tissue remains “quiescent” due to insufficient excitation energy, thereby naturally suppressing background noise. This mechanism not only substantially reduces the optical and mechanical complexity of the system but also enables MUSE to acquire structural clarity comparable to, or even superior to, traditional frozen sections at wide-field imaging speeds. However, this physical constraint also implies that imaging information is strictly limited to the specimen surface; consequently, surface flatness during sample preparation and subsequent computational processing are critical to final image quality.

#### 3.1.2. Signal Enhancement

The histological semantic interpretation of MUSE hinges upon the complementary contrast of multi-channel fluorescence signals. These signals primarily originate from two sources. The first is endogenous autofluorescence, such as the specific fluorescence generated by collagen, elastin, and metabolic coenzymes (NADH/FAD) under UV excitation, which provides natural contrast for the tissue matrix and metabolic state. The second is exogenous rapid surface staining, which is key to achieving the “H&E-like” effect in MUSE. Leveraging the high surface confinement of UV excitation, MUSE employs fluorescent dyes with high quantum yields (e.g., Hoechst, eosin, or rhodamine) for surface treatment lasting from seconds to minutes. This staining strategy follows a “stain-and-image” engineering philosophy: dyes need only bind to superficial cellular structures, eliminating the need for deep penetration required in traditional pathological staining [17]. This distinction is important when describing MUSE within a sensing-oriented review. In its strictest form, MUSE is not always purely label-free. Rather, its main advantage is that it is slide-free and preparation-light, and that even when staining is used, the staining process is rapid, superficial, and workflow-compatible. Multispectral acquisition separates emission light from different bands (e.g., the blue channel corresponds to cell nuclei, while green/red channels correspond to cytoplasm and matrix), translating biochemical compositional differences into color space variations. Subsequent studies have demonstrated that this mechanism, based on physically restricted excitation combined with multispectral chemical enhancement, can stably output morphological features highly consistent with standard pathology, laying a solid informational foundation for generating virtual H&E images directly from ex vivo tissues [42,43].

While the physics of surface-confined UV excitation establishes the feasibility of slide-free histology, the translational value of MUSE ultimately depends on whether system implementations satisfy workflow constraints—particularly staining time, field of view, and time-to-image in intraoperative and point-of-care settings.

### 3.2. Engineering Evolution and Clinical Use-Cases

Having established the physical principles of MUSE, the pivot from laboratory prototyping to clinical application hinges on resolving the contradictions between engineering robustness, imaging throughput, and workflow compatibility. Unlike traditional super-resolution microscopy, which pursues extreme resolution, the engineering philosophy of MUSE is workflow-driven: it aims to minimize sample preparation complexity and system hardware costs to provide images of pathological diagnostic value within a minute-level time window. This approach overcomes the temporal bottlenecks of traditional H&E preparation in rapid intraoperative assessment and low-resource settings [13,17]. This section explores the evolutionary path of MUSE system engineering, analyzes its clinical niche in dermatopathology and breast pathology, and specifically discusses its potential in portability and multimodal fusion. 

#### 3.2.1. Hardware Evolution and System Innovation

Early MUSE prototypes established the feasibility of slide-free, UV surface excitation histology, but real clinical specimens quickly exposed system-level bottlenecks. First, tissue surfaces are rarely planar, so wide-field imaging is prone to spatially varying defocus that directly degrades nuclear detail and reduces diagnostic reliability. Second, deep UV illumination below 300 nm is poorly compatible with common optical glasses, which increases reliance on quartz or other UV transmissive components and raises system cost. At the same time, conventional dry objectives and non-optimized detection chains can limit fluorescence collection, so signal to noise becomes a practical constraint rather than a purely photophysical issue.

**Figure 5:**
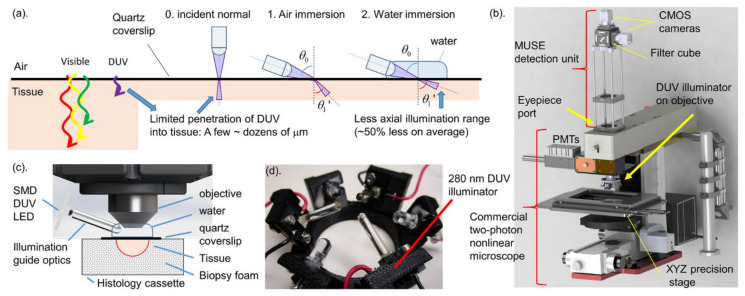
Engineering implementation of MUSE: immersion optics and compact architecture. (**a**) Principle of immersion optics: comparison of optical paths between air and water immersion modes. The water medium improves the refraction angle at the tissue interface, significantly enhancing the NA and fluorescence collection efficiency. (**b**,**d**) Compact hardware integration: (**b**) shows the vertical detection unit integrating CMOS cameras, filter cubes, and precision stages; (**d**) utilizes a miniaturized 280 nm UV LED ring array as the excitation source, replacing bulky laser systems and establishing the foundation for box-type instruments. (**c**) Detail of the immersion flattening interface: uses a quartz coverslip with a water medium to create a liquid interface, physically flattening the tissue while maintaining optical contact to resolve defocusing issues caused by uneven tissue surfaces. (Reprinted from ref. [43]).

**Figure 6:**
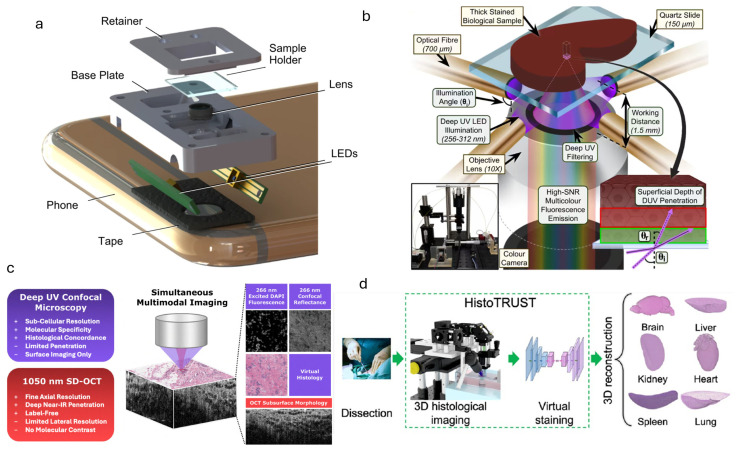
Morphological evolution of MUSE technology: from portable terminals to multimodal fusion systems. (**a**) Portable: pocket MUSE utilizes smartphones, 3D-printed attachments, and consumer-grade LEDs to drastically reduce hardware costs for point-of-care screening in low-resource settings [47]. (**b**) Endoscopic: a fiber-optic-based MUSE architecture that extends imaging capabilities to in vivo cavities while retaining the UV surface excitation mechanism [48]. (**c**) Multimodal: a synergistic system combining deep UV surface imaging with optical coherence tomography (OCT) to provide complementary cell-specific morphology and deep tissue structural information [49]. (**d**) Volumetric: HistoTRUST technology enables rapid 3D color pathological reconstruction at the whole-organ scale through a “surface imaging + automated sectioning” strategy [10]. Panels (**a**–**d**) are adapted from refs. [47], [48], [49], and [10], respectively.

A key engineering turning point was the introduction of immersion-based optical designs and liquid interface strategies. Ching Roa et al. showed that a liquid interface can improve tolerance to modest tissue topography by stabilizing the focal condition while also increasing effective numerical aperture and fluorescence collection efficiency. This improves image consistency across uneven samples and reduces the dependence on high power UV sources, which is important for compact and cost-controlled implementations [44]. Figure 5 summarizes the immersion optics principle, the liquid contact-flattening interface, and the compact integration of deep UV illumination with CMOS-based detection that enables robust wide-field scanning. As deep UV LEDs matured, including the wider availability and improved packaging of 275 nm and 285 nm devices, and as CMOS sensor performance improved, MUSE systems increasingly shifted away from research microscope form factors toward compact box type instruments and handheld derivatives (Figure 6a). In these implementations, the hardware workflow becomes standardized around rapid surface staining, immersion-assisted flattening, and wide-field digital scanning, which together define a practical engineering paradigm for minute-scale histology-like readout [43].

Clinical deployment also imposes throughput requirements that are not solved by optics alone. This drove integration-oriented designs where UV LED arrays, filters, and CMOS detection are packaged into stable modules with controlled geometry, reducing alignment burden and improving day to day robustness. For large specimens, automated translation stages and wide-field mosaicking algorithms enable large area digitization and gigapixel reconstruction, which is essential for surgical margin workflows that require both coverage and microscopic detail [45]. In parallel, MUSE followed a second trajectory emphasizing accessibility. Pocket MUSE illustrates how consumer-grade UV LEDs, 3D-printed mechanical adapters, and smartphone-based imaging and computation can compress cost and complexity, enabling point-of-care use and educational deployment, and aligning with global health settings where conventional pathology infrastructure is limited [33,39,46].

More recent hardware innovation has focused on extending MUSE beyond the traditional constraints of ex vivo operation and surface only readout. Fiber bundle-based implementations provide a practical route to deliver UV excitation and collect wide-field fluorescence in endoscopic geometries, expanding the platform toward luminal and anatomically constrained scenarios(Figure 6b) [48]. To compensate for the lack of depth information, combined MUSE and OCT systems integrate cellular level surface contrast with depth resolved structural context, improving interpretability when subsurface architecture matters (Figure 6c) [49]. For volumetric histology, serial imaging approaches such as TRUST combine UV excitation with sectioning and repeated acquisition to build centimeter scale, multicolor 3D datasets, demonstrating that whole organ atlas generation can be achieved with relatively accessible hardware when the workflow is engineered as an integrated system (Figure 6d) [10,50].

#### 3.2.2. Representative Applications

**Figure 7:**
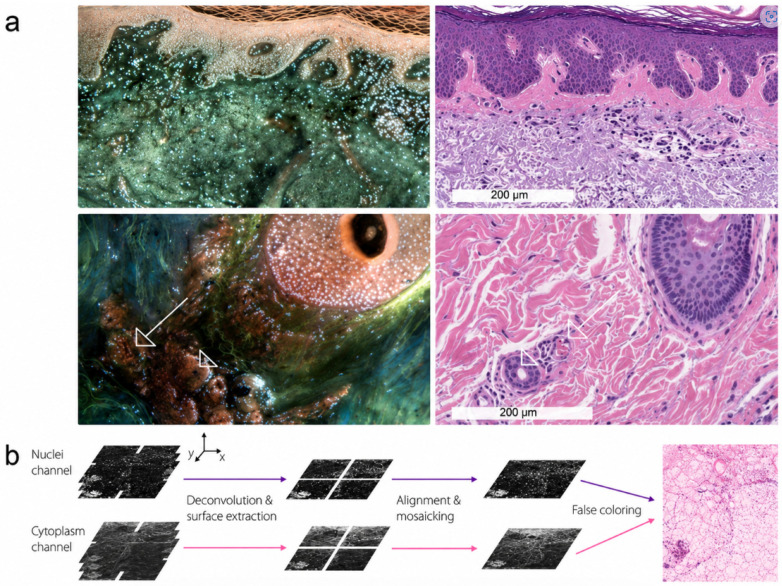
Clinical validation and image reconstruction workflow of MUSE. (**a**) Morphological comparison in skin tissue: MUSE pseudo-colored images (**left**) show high consistency with standard H&E sections (**right**) in identifying epidermal-dermal junctions and appendages. (**b**) Wide-field image processing workflow: the pipeline involves dual-channel acquisition, surface extraction, image mosaicking, and false coloring to rapidly generate large-area histological images. (Adapted from refs. [42,45]).

With these hardware capabilities in place, early clinical applications of MUSE concentrate in scenarios where superficial imaging, rapid turnaround, and H&E familiar visualization offer immediate practical advantage. Dermatopathology is a natural entry point, particularly in settings such as Mohs micrographic surgery where surgeons require fast, reliable determination of tumor margins and where frozen sections can be time consuming and artifact prone. By exploiting shallow UV penetration, MUSE emphasizes nuclear distribution and microstructural features near the epidermal dermal junction and adnexal structures that are central to diagnosing common skin malignancies. Qorbani et al. reported that after pseudo color processing, MUSE images can reproduce key diagnostic features of standard H&E with greater than 90 percent concordance, while maintaining a workflow on the order of minutes, which supports intraoperative decision making and rapid screening [42]. Figure 7a shows the morphological concordance between pseudo colored MUSE images and standard H&E sections in skin tissue, highlighting epidermal dermal junctions and adnexal structures relevant to dermatopathology.

Breast tumor margin assessment represents a second high value niche because it places simultaneous demands on field of view and throughput. Fat rich breast tissue is difficult to cryosection and often fragments, so the slide-free and no sectioning nature of MUSE directly addresses a major sample preparation barrier. In this context, the engineering emphasis shifts toward wide area scanning and image mosaicking that preserve both macroscopic coverage and nuclear level detail. Xie et al. demonstrated wide-field MUSE capable of imaging margin tissues across several square centimeters and reconstructing gigapixel scale mosaics, enabling cross scale review from overview to cellular morphology and supporting high-throughput intraoperative margin screening [45]. Figure 7b summarizes the wide-field reconstruction workflow for large specimens, including dual channel acquisition, surface extraction, alignment, mosaicking, and false coloring to rapidly generate large-area histology-like images.

Low-resource and point-of-care settings further highlight the same logic of rapid, preparation minimal histology-like imaging. Portable and ultra-low-cost implementations such as Pocket MUSE use smartphone optics and computation to provide histology style visualization outside conventional laboratory environments, offering a plausible pathway for decentralized screening and training where access to standard pathology services is limited [33,39,46]. Representative MUSE system configurations, workflow characteristics, and clinical translation features are summarized in Table 4.

### 3.3. AI-Based MUSE

With the physical and engineering foundations of MUSE for acquiring histological information within minutes well established, the integration of AI has begun to assume a pivotal role as a bridge from “imaging” to “decision-making.” This involves lowering cognitive barriers via virtual staining to mitigate modality differences, enhancing robustness under multi-center deployment and real-world sample conditions through quality normalization and whole-slide reconstruction, and ultimately extending to task-oriented CAD to form a human-in-the-loop collaborative cycle embedded within clinical workflows [13,17], Table 5 summarizes the AI applications within MUSE technology.

#### 3.3.1. Virtual Staining

Similar to the challenges faced by UV-PAM (see Section 2.3.1), MUSE must bridge the visual discrepancy between fluorescence pseudo-colors and standard H&E staining. Style transfer architectures based on unpaired Generative Adversarial Networks (e.g., CycleGAN) are widely adopted to circumvent the registration difficulties caused by physical sectioning deformations [52].

However, unlike the single-channel grayscale input of UV-PAM, virtual staining in MUSE possesses unique spectral mapping characteristics. Since raw MUSE images contain independent fluorescence information for nuclei (blue channel) and cytoplasm/stroma (green or yellow channel), the core task for the AI model is to establish a non-linear mapping relationship between multi-channel spectral combinations and H&E dye absorbance. Chen et al. demonstrated that this multi-channel input strategy not only achieves alignment in color space but also maintains high pathological consistency in nuclear texture and stromal contrast [51]. Recent studies further indicate that deeply fusing rich information from the spectral dimension can significantly enhance the specificity of virtual staining, effectively mitigating the risks of style transfer hallucinations and distortions common in complex tissue types, thereby ensuring fidelity in pathological semantics [53].

#### 3.3.2. Domain Adaptation

As MUSE transitions from the laboratory to multi-center clinical deployment, domain shift emerges as a non-negligible engineering challenge. Variations in UV source intensity decay, spectral differences in filters, noise responses of camera sensors, and operator techniques across different batches can lead to significant color and brightness drifts in raw images. Such inconsistencies not only interfere with clinician interpretation but also severely degrade the generalization capability of downstream automated analysis algorithms.

In this context, AI is assigned the new role of quality normalization. By training denoising and illumination correction models, heterogeneous data acquired from different devices can be mapped to a unified, standardized feature space. Research by Chen et al. points out that deep learning models can perform enhancement processing simultaneously with image translation, automatically correcting artifacts caused by uneven illumination or rapid scanning, thus significantly improving image readability [51].

More representatively, in “hardware-constrained” scenarios such as endoscopy and fiber-optic image transmission, quality consistency issues manifest as structural artifacts and information loss. Ang et al. observed in a fiber-based MUSE system that the inherent pixelation effects of fiber bundles disrupt nuclear texture and boundary continuity, affecting the reliable presentation of histological semantics. Their work demonstrated the feasibility of using generative models to repair fiber pixelation artifacts and enhance quality, resulting in output images that closely approximate standard MUSE morphological performance in terms of texture continuity and visual consistency [48]. From a broader clinical translation perspective, such AI strategies combining cross-device consistency and artifact correction align highly with pathological requirements for reproducibility and quality control, serving as a critical condition for MUSE to pass regulatory approval and achieve scalable clinical adoption [17].

#### 3.3.3. Robustness Enhancement

Although MUSE relies on surface flatness, real biological tissues (especially fresh specimens without cryo-embedding) inevitably exhibit micron-scale undulations. Under the high numerical aperture of wide-field microscopy, such undulations easily cause regions to fall out of the focal plane (defocus), leading to a loss of diagnostic information. EDOF technology has thus become a key component in enhancing the robustness of MUSE, primarily following two routes: pure algorithmic approaches and hardware-software synergy.

The pure algorithmic route typically employs focus stacking or single-image deep learning deblurring. Dogan et al. constructed a DL-EDOF dataset and method specifically for microscopy, proving that deep networks can recover clear textures from images containing defocus blur, thereby relaxing the physical requirements for sample flatness [54]. The other route involves the synergy of optical hardware and computational reconstruction. Jeon et al. proposed using a micromirror array system to achieve rapid varifocal or multi-view acquisition, combined with computational imaging algorithms to reconstruct all-in-focus images [55]. Although this “hardware encoding combined with software decoding” strategy increases system complexity, it offers more reliable physical constraints than pure end-to-end generative models when dealing with samples having large undulations, providing a feasible solution for complete imaging of large, non-flat tissues (e.g., surfaces of resection specimens). While these EDOF works may not be exclusive to MUSE, their generalizability to wide-field microscopy systems makes them transferable solutions for enhancing MUSE engineering robustness.

#### 3.3.4. Decision Support

The ultimate goal of combining MUSE with AI is not merely to generate an “aesthetically pleasing” H&E-like image, but to provide “actionable” decision support. Future research emphasis will shift from image generation to CAD, utilizing segmentation and classification networks to directly extract clinically relevant metrics, such as nuclear counts, nuclear atypia scores, tumor region detection, and margin positivity alerts.

Work by Chen et al. has preliminarily demonstrated the potential for automated analysis on virtually stained images [51]; however, achieving genuine clinical implementation requires addressing compliance and generalizability issues. Richards-Kortum et al. emphasized that screening tools in low-resource settings must possess extremely high degrees of automation and robustness to reduce reliance on specialized pathologists [46]. From a surgical pathology perspective, future AI-MUSE systems should be capable of human interaction: AI serves to rapidly prescreen and highlight suspicious areas (e.g., micro-invasion foci), while the pathologist is responsible for final confirmation, thereby forming an efficient human-machine collaborative workflow [17]. The roadmap ahead should include constructing large-scale, multi-center MUSE datasets covering diverse tissue types and establishing assessment metrics oriented toward clinical outcomes (e.g., sensitivity/specificity of margin assessment), thereby elevating AI from an image enhancement tool to a trustworthy diagnostic companion.

### 3.4. Challenges and Future

UV fluorescence imaging and MUSE-like approaches can provide rapid, slide-free tissue assessment, but their reliability remains sensitive to specimen preparation and workflow boundary conditions, including surface topography, hydration, staining and rinsing steps, photobleaching, and fluorescence collection consistency. These factors can introduce substantial site-to-site and operator-to-operator variability, reducing generalization across laboratories and clinical settings. Accordingly, the translational challenge of MUSE is not only to produce histology-like images quickly but also to do so reproducibly under realistic workflow constraints.

When deep learning is used for virtual staining, image restoration, or modality translation, additional risks arise from hallucinated structures, feature distortion, and degraded performance under domain shift. For this reason, AI-assisted MUSE systems should be evaluated not only by image similarity metrics but also by uncertainty reporting, preservation of diagnostically relevant features, and task-based validation such as diagnostic concordance, sensitivity, specificity, and inter-reader agreement. Clinical adoption will also depend on standardized specimen handling, time-to-result, integration with pathology workflows, and evidence that aligns with regulatory expectations. Future work should prioritize cross-center validation, constrained model design, and protocol standardization so that MUSE can move from promising rapid imaging to trustworthy pathology support. More broadly, recent reviews of ultraviolet microscopy indicate that future progress in UV surface-weighted imaging will likely rely on better UV source integration, workflow-aware system design, and AI-assisted interpretation rather than on optical hardware optimization alone [11].

## 4. Wearable and Embedded UV Dosimetry

**Figure 8:**
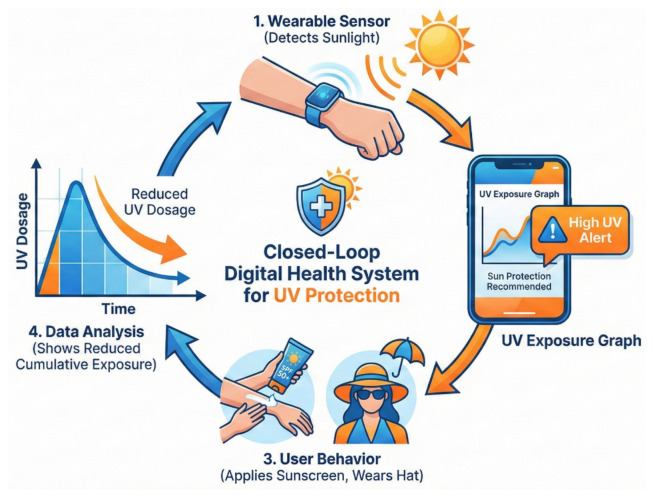
Feedback-informed workflow of wearable UV dosimetry.

Wearable and embedded dosimeters turn UV exposure into a measurable dose at the skin level. Within the broader framework of this review, this capability is important because dosimetry provides the most direct bridge between irradiation, biological risk, and quantitative decision-making. This step supports calibration, safety limits, and feedback-informed management when it is combined with imaging readouts and clinical outcomes. In Section 2 and Section 3, we discussed UV light as an active probe used to excite photoacoustic or fluorescent signals for cellular and tissue-level diagnosis. However, UV radiation possesses a typical duality in biomedical engineering: it serves not only as an energy carrier for imaging and therapy but also constitutes a significant environmental exposure factor contributing to photoaging, DNA damage, and skin cancer risk. Consequently, extending from active excitation imaging to passive exposure monitoring to establish a precise, human-oriented UV dosimetry system is a critical link in constructing a closed-loop ecosystem of diagnosis, prevention, and treatment (Figure 8). Unlike benchtop radiometers, wearable and embedded UV dosimetry must simultaneously satisfy engineering constraints such as skin conformability, low power consumption or passive operation, long-term wearability, and quantifiable readout. More importantly, these systems are valuable not simply because they detect UV but because they attempt to estimate the true dose received at the tissue interface under conditions where ambient UV, device settings, and nominal exposure often differ substantially from biologically relevant dose. These requirements have driven the synergistic evolution of materials, device structures, and system architectures [9,56].

### 4.1. Sensing Mechanisms and Engineering Basis

The core challenge in UV dosimetry for real-world scenarios lies not merely in photodetection, but in maintaining traceable and comparable dosage metrics under conditions of severe skin deformation, sweat/oil contamination, and rapidly changing incident angles. Existing technical routes can be broadly categorized into three types: photochemical colorimetry (passive accumulation), photoelectronic detection (real-time electrical signal), and flexible mechanics (conformal contact to mitigate motion artifacts and cosine errors).

#### 4.1.1. Photo-Chemical/Colorimetric Approaches

The primary advantage of photochemical colorimetric strategies is their passive nature: UV light directly drives molecular configuration changes or irreversible reactions, mapping invisible UV exposure to visible color changes to record accumulated dosage. A typical implementation involves encapsulating UV-selective colorimetric chemical systems into thin films and establishing a quantitative relationship between color/optical density and dosage through calibration.

**Figure 9:**
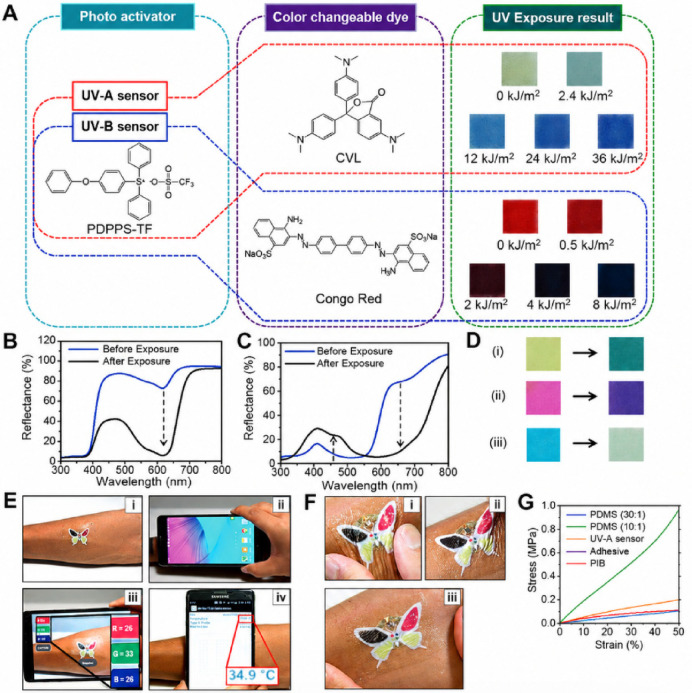
NFC-enabled epidermal UV colorimetric dosimeter. (**A**) Schematic of the UV-A/UV-B sensing chemistry based on photoacid activation and pH-sensitive dyes (PDPPS-TF combined with CVL or Congo Red), where the colored dashed lines and arrows indicate the respective chemical sensing workflows and subsequent dose-dependent color changes. (**B**,**C**) Reflectance spectra before and after UV exposure. (**D**) Representative color transitions for (i) the UV-A sensor, (ii) the UV-B sensor, and (iii) the co-loaded sensing matrix. (**E**) Smartphone-based color readout and NFC-powered operation showing: (i) the flexible patch attached to the forearm, (ii) approaching the device with an NFC-enabled smartphone, (iii) real-time digital colorimetric RGB value mapping via a custom app, and (iv) regional skin temperature readout (34.9 °C). (**F**) On-skin conformability of the flexible patch under different mechanical deformations: (i) relaxed, (ii) pinched, and (iii) twisted states, demonstrating excellent mechanical compliance. (**G**) Stress–strain curves of device materials including PDMS, adhesive, and PIB. (Reprinted with permission from ref. [57]. Copyright 2024 The Royal Society of Chemistry).

Representative work comes from the Rogers group: Araki et al. proposed an epidermal UV colorimetric dosimeter coupled with near-field communication (NFC) (Figure 9). This patch allows for both coarse visual estimation by the naked eye and digital readout/recording via terminals like smartphones, effectively upgrading the “cheap disposable chemical dosimeter” into a scalable node for individual exposure monitoring [56]. The engineering crux of such solutions lies not in the single chemical reaction, but in the low-modulus mechanical matching of the encapsulation with skin and the traceability of the readout (e.g., NFC standardization), thereby supporting more reliable behavioral interventions and epidemiological data collection at the population level [56].

#### 4.1.2. Photo-Electronic Approaches

**Figure 10:**
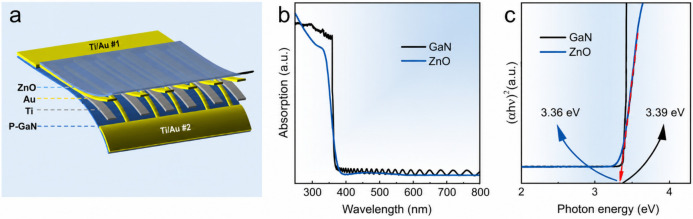
Flexible self-powered UV photodetector based on wide-bandgap semiconductor heterojunctions. (**a**) Schematic of the device structure: a heterojunction is constructed using ZnO and p-GaN, where the built-in electric field at the interface facilitates effective separation of photo-generated carriers, enabling a self-powered response under zero bias. (**b**,**c**) Optical characterization: (**b**) absorption spectra demonstrate strong absorption in the UV region and transparency in the visible spectrum, validating its resistance to visible light interference; (**c**) bandgap analysis confirms the wide bandgap nature (~3.36–3.39 eV), establishing the physical basis for the device’s “intrinsic selectivity” for UV detection. In subfigure (**a**), “#1” and “#2” denote the two Ti/Au electrode contacts. (Reprinted with permission from ref. [56]. Copyright 2017 Wiley-VCH Verlag GmbH & Co. KGaA).

In contrast to colorimetric methods that focus on recording accumulated exposure, photoelectronic UV sensors utilize current or voltage signals as direct outputs, making them more suitable for resolving transient irradiance fluctuations caused by cloud cover or movement through shade. To suppress visible light crosstalk and enhance the signal-to-noise ratio (SNR), flexible UV sensors typically employ wide-bandgap semiconductors (e.g., ZnO, GaN, and Ga_2_O_3_), achieving intrinsic selectivity for UV light at the band structure level. Addressing the energy constraints of long-term wearability, self-powered UV detection has emerged as a significant development direction. By introducing built-in electric fields through heterojunctions or Schottky barriers, effective separation of photo-generated carriers can be achieved under zero external bias, enabling self-driven response. Taking the ZnO/p-GaN composite device reported by Chen et al. as an example, the synergistic design of multi-junction built-in fields realizes rapid response and a high light-to-dark ratio under zero bias. Figure 10 verifies the intrinsic selectivity and real-time digital output capability of UV detection from the perspective of device physics [57]. It is important to note that “self-powered” does not imply the system is entirely free of energy management; rather, it shifts energy consumption from continuous power supply to intermittent communication and edge computing, aligning better with the system-level requirements of long-term, low-burden wearable applications [57].

#### 4.1.3. Mechanics and Conformal Contact

Accurate measurement of UV exposure is highly dependent on incident geometry. When warping, delamination, or air gaps occur between the sensor and the skin, changes in the incident angle introduce significant cosine errors, causing the recorded dosage to deviate from the actual skin received dosage. Consequently, dosimetry is never a purely optical problem, but a coupled problem involving the optics-mechanics-materials interface.

**Figure 11:**
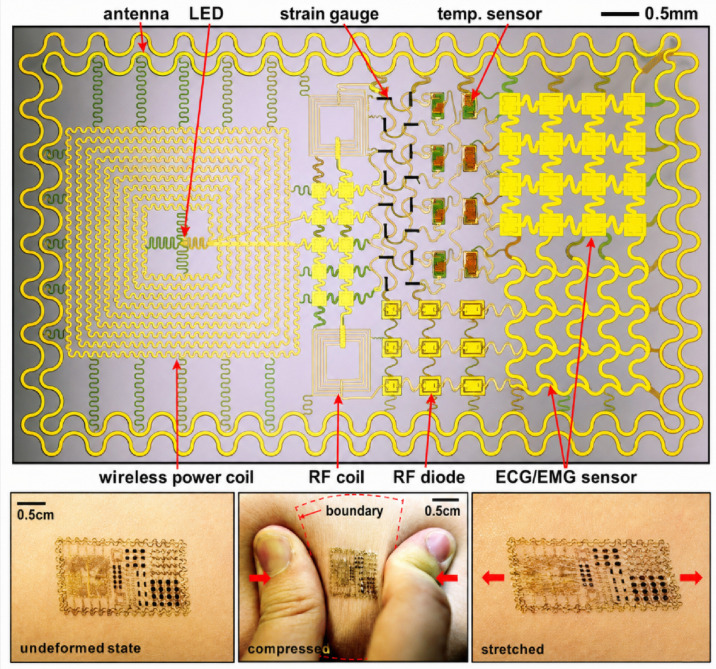
Epidermal electronic system based on “island-bridge” structure and conformal contact mechanics. (**Top**) Device microstructure: serpentine interconnects (“bridges”) connect rigid functional islands (e.g., wireless coils, LEDs, sensors), achieving a macroscopically low equivalent modulus matched to skin. (**Bottom**) On-skin integration: demonstrates that the device maintains tight conformal contact with the skin surface under undeformed, compressed, and stretched states. This mechanical compliance effectively eliminates air gaps, serving as a critical engineering paradigm to physically mitigate cosine errors in UV dosimetry. (Reprinted with permission from ref. [58]. Copyright 2011 American Association for the Advancement of Science).

The island–bridge architecture proposed by “epidermal electronics” provides a reusable mechanical paradigm for integrating high-performance devices on soft tissue surfaces. This approach miniaturizes rigid functional units into “islands” and uses serpentine interconnects as “bridges” to absorb deformation, thereby achieving low equivalent modulus and high conformal adhesion at the macroscopic level. As shown in Figure 11, this structure maintains intimate contact and avoids interfacial voids under undeformed, compressed, and stretched states. From an engineering perspective, this mitigates incident angle errors caused by postural changes and poor adhesion, providing a measurement prerequisite for UV dosimeters that is closer to “actual exposure” [58]. For UV dosimetry, the significance of this paradigm lies in elevating “adhesion stability” to a metrological prerequisite, bringing wearable dosage data closer to the quality levels required for clinical and public health decision-making [58].

Representative wearable and embedded UV dosimetry approaches, including their sensing principles, power strategies, spectral selectivity, angular correction, skin conformality, outputs, application scenarios, and key fidelity limitations, are summarized in Table 6.

### 4.2. Application Evolution

Wearable UV dosimetry has evolved primarily as an application-driven technology that closes the gap between nominal or environmental UV metrics and the actual dose delivered to skin in real settings. Early systems were adopted first in public health and prevention, where the key need is to quantify cumulative exposure along individual behavior trajectories rather than to achieve clinical-grade absolute accuracy; by turning “personal exposure” into a measurable variable, wearable sensors enable risk awareness, evaluation of sun-protection interventions, and management of high-risk populations. A systematic review of wearable UV sensors highlighted that devices vary widely in output format (real-time versus cumulative), accuracy, compliance, and cost but share a common practical value: making individualized exposure data accessible for behavioral assessment and prevention strategies [19]. Building on this, field studies have demonstrated deployable workflows that integrate dosimeters with mobile platforms to deliver real-time feedback and prompts based on measured exposure, providing a feasible route to data-driven behavior change in daily life [62], and randomized evidence further suggests that UV sensors can complement conventional sun-protection education as a behavioral modification tool [61]. With improved device stability, skin conformity, and system integration, the application focus of wearable UV dosimetry has expanded from prevention into clinical phototherapy. In this setting, the main requirement shifts from general exposure awareness to dose traceability and skin-level accuracy, because nominal device settings do not reliably represent the dose actually delivered to treatment sites under conditions of variable positioning, source-to-skin distance, partial occlusion, and body-surface curvature. Epidermal and skin-mounted dosimeters therefore become precision companions rather than simple exposure badges: by moving the measurement point to the skin interface, they support calibration of delivered dose and more individualized regulation of treatment workflows, as illustrated by representative approaches in Table 6. A representative example is the wireless, battery-free, flexible micro-dosimeter platform that can synchronously monitor natural solar radiation and clinical phototherapy light, demonstrating real-time and cumulative dose tracking while also establishing a transferable system concept that can be adapted across wavelengths and therapeutic contexts [9]. More broadly, the evolution of wearable UV dosimetry points toward a longer-term role in personalized exposure and treatment management. The key issue is no longer whether UV can be detected but whether measured dose can be interpreted in a way that remains meaningful across changing biological, environmental, and clinical conditions. In this sense, wearable UV dosimetry is becoming not just a device category but a metrological and decision-support layer for UV-enabled biomedical systems. Its major advantage is that it shifts dose assessment from ambient proxies or nominal source settings toward direct, individualized, skin-level measurement. Its main challenge is that measured sensor output is not automatically equivalent to biologically effective dose. Instead, accurate interpretation requires correction for spectral mismatch, incidence angle, sensor placement, skin conformity, environmental drift, and inter-individual variability. To make this shared workflow explicit across Section 2, Section 3, Section 4 and Section 5, Table 7 provides a cross-platform synthesis of UV-PAM, MUSE, wearable and embedded dosimetry, and UV phototherapy in terms of target, transduction, calibration needs, main uncertainty sources, and decision output. Looking ahead, the same application logic suggests that future systems will increasingly combine multimodal sensing with computational analysis to support longer-term personalized exposure management and therapy supervision, while remaining grounded in the metrological reality that neither meteorology-based UV Index nor device-defined irradiation parameters can fully represent true skin-level dose in specific real-world contexts.

### 4.3. AI-Enabled Dosimetry

Wearable UV dosimetry is increasingly used as a practical tool for exposure management, where the goal is to convert continuous measurements into timely, individualized guidance rather than merely reporting irradiance or cumulative dose. In this context, the value of artificial intelligence lies not simply in improving prediction accuracy but in helping translate raw or partially corrected sensor outputs into decision-relevant estimates of tissue-relevant exposure. For users and clinicians, the most meaningful outputs are decision-oriented endpoints, such as erythema risk, DNA damage probability, and an effective phototherapeutic window, rather than irradiance or cumulative dose alone. These endpoints cannot usually be inferred from a sensor signal in a direct or universal manner, because the relationship between measured UV and biologically effective dose depends on multiple interacting factors, including spectral composition, incidence geometry, anatomical site, skin phenotype, prior exposure history, and user behavior. In this setting, AI does not replace sensors; rather, it functions as a computational correction and interpretation layer that helps connect measured UV exposure to clinically or behaviorally actionable estimates by accounting for individual factors and measurement uncertainties across geometry and time [63,64]. Regarding individual differences, traditional approaches such as Fitzpatrick skin typing are subjective and have limited cross-population generalizability; evidence of performance degradation and bias in dermatological AI for darker skin tones further highlights the need for diverse training data and calibration to improve fairness and reliability when building individualized risk models [65]. For UV dosimetry, this means that AI models should not rely solely on population-average assumptions but should instead improve the interpretation of measured exposure under heterogeneous biological conditions. Beyond phenotype, the problem is not only who is being measured but also how and where the measurement is made. Natural pose changes alter the angle between the sensor normal and solar incidence, producing cosine-response deviations, and pose variation has been repeatedly identified as a primary contributor to error in personal exposure measurement [18,19]. To reduce these errors without complex optical redesign, recent systems integrate multi-axis inertial measurement units with machine learning to correct readings using pose, temporal, and geographic information, thereby positioning AI as a practical physical-compensation module embedded within dosimetry rather than as a purely post hoc analytical tool [64].

At the same time, real-world exposure is highly dynamic because of changing solar angle, cloud cover, transient occlusion, daily activity patterns, and time-dependent sunscreen degradation. These fluctuations make passive dose logging insufficient when the intended goal is prevention, early warning, or therapy supervision. AI therefore supports a shift from passive logging to prediction and intervention. The dosimeter–smart terminal–feedback paradigm illustrates how sensor-derived exposure can be converted into real-time behavioral guidance through algorithmic or rule-based decision modules, while user responses in turn shape subsequent exposure trajectories, enabling continuous exposure management and building dose–behavior–outcome datasets that can support more individualized intervention strategies [62]. Implementation typically relies on time-series modeling, where recurrent neural networks or state space models use historical UV dose together with environmental variables (time, location, cloud cover) and behavioral states to forecast near-term exposure risk [66]. Within this framework, the role of AI is not limited to forecasting future UV levels but extends to estimating whether an observed exposure pattern is likely to exceed a safety threshold or drift outside a clinically acceptable treatment window.

Emerging work further explores reinforcement learning or contextual optimization to minimize cumulative risk or the probability of exceeding safety thresholds for dynamically updated, individualized protection or treatment strategies [67,68]. However, these more advanced decision models should currently be interpreted as emerging and supportive rather than as fully validated closed-loop control solutions. Their translational value will depend on whether they can operate under realistic uncertainty, maintain robustness across users and environments, and remain interpretable enough for preventive or clinical use. More broadly, the development of AI-enabled dosimetry should be understood as part of a larger shift from UV measurement alone toward dose interpretation, uncertainty-aware risk estimation, and feedback-informed exposure management. In that sense, AI contributes most credibly when it improves calibration, personalization, and actionability around measured dose, rather than when it is presented as a standalone replacement for sensing or clinical judgment.

### 4.4. Challenges and Future

Wearable UV dosimetry must deliver accurate, interpretable dose estimates during long-term use in uncontrolled environments. Its main strength is that it can move UV assessment from ambient or nominal exposure toward individualized, skin-level measurement. Its main weakness is that this translation is technically difficult and often incomplete. Sensor readings may drift with temperature, aging, hydration, and mechanical deformation, and they are strongly affected by spectral mismatch, incidence angle, placement variability, and incomplete skin conformity. In addition, biological modifiers such as skin tone, stratum corneum properties, sweating, cosmetics, sunscreen use, and lesion state can weaken the relationship between measured physical dose and biologically effective dose.

These limitations show that the central problem in wearable UV dosimetry is not detection alone but metrological validity. A clinically useful system must specify what kind of dose is being estimated, under what assumptions, and with what uncertainty. At present, unified conventions for band separation, erythema weighting, integration time, calibration traceability, and uncertainty reporting remain limited across the field. Longitudinal datasets linking wearable UV dose to clinical outcomes are also still sparse. Recent spectral characterization studies of wearable electronic UV dosimeters further show that devices can differ substantially in spectral responsivity, reinforcing the need for explicit characterization and calibration rather than assuming equivalence across platforms [69].

Future progress will likely depend on combining three elements: first, traceable calibration procedures and standardized reporting; second, multimodal sensing that integrates UV measurements with posture, environment, and skin state; and third, AI-assisted correction models that convert imperfect physical measurements into more individualized and decision-relevant dose estimates. The most useful next-generation dosimetry systems will therefore be those that do not merely record UV exposure, but that translate dose into uncertainty-aware risk assessment, treatment supervision, or prevention guidance.

## 5. Sensor-Informed and Dose-Guided UV Phototherapy

UV radiation is also widely used as a therapeutic or preventive input. In this context, the relevance of sensing lies not in redefining phototherapy itself as a canonical biosensing modality, but in improving how therapeutic UV delivery is measured, calibrated, interpreted, and adjusted in practice. Building on the diagnostic readouts discussed in Section 2 and Section 3 and the quantitative dose information summarized in Section 4, UV therapy can increasingly be examined as a sensing-assisted therapeutic domain in which dosimetry, imaging, and computational analysis help reduce the gap between nominal irradiation settings and biologically effective dose.

Importantly, however, most current UV therapeutic workflows remain predominantly open loop. In routine clinical and preventive use, dose is still commonly prescribed according to device settings, exposure time, or empirical escalation rules, with limited real-time feedback on target-site dose and tissue response. From an engineering perspective, the main challenge in UV phototherapy is therefore not only the generation of therapeutic irradiance, but also the achievement of targeted, quantifiable, and safety-constrained dose delivery under conditions of strong tissue heterogeneity, variable geometry, and narrow therapeutic windows.

**Figure 12:**
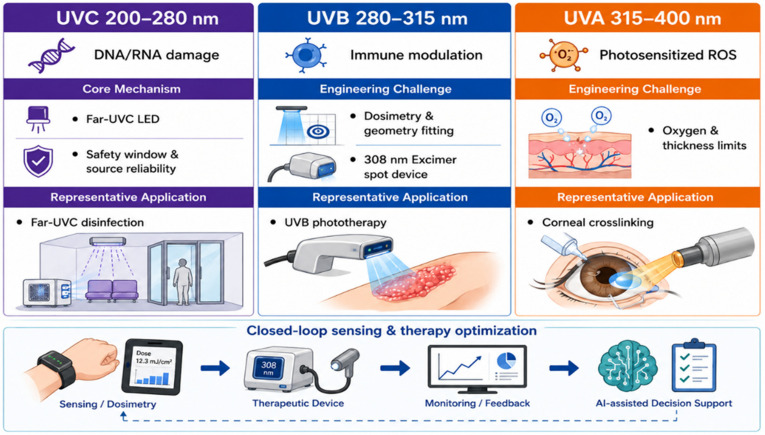
Ultraviolet-assisted phototherapy organized by wavelength (200–400 nm).

Accordingly, this section reviews UVC, UVB, and UVA systems by wavelength while paying particular attention to where sensing and quantitative feedback are already established, where they remain partial, and where they are still mainly conceptual. In this sense, the systems discussed below should be interpreted as sensing-assisted or biosensing-adjacent therapeutic platforms rather than uniformly mature closed-loop treatment systems. The wavelength-organized framework of ultraviolet-assisted phototherapy and its sensing-assisted optimization workflow are summarized in Figure 12.

### 5.1. UVC (200–280 nm)

#### 5.1.1. Physical Mechanism

**Figure 13:**
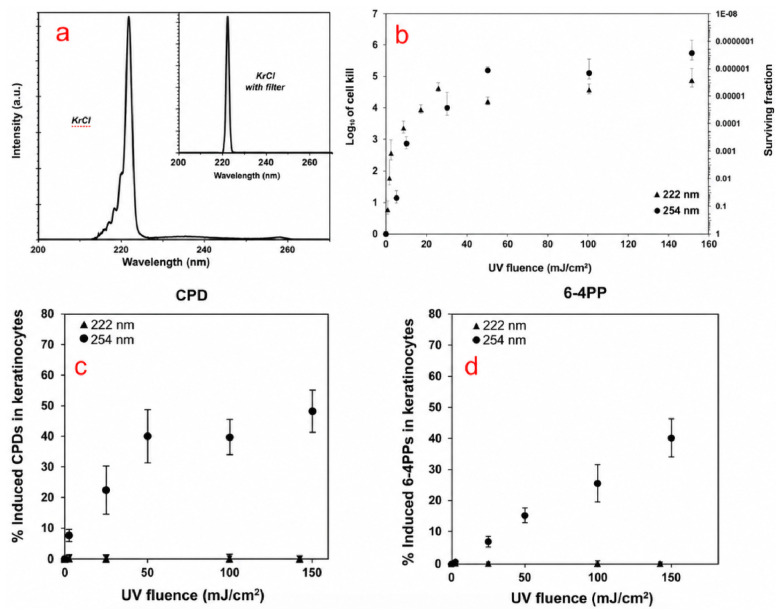
Spectral output, germicidal efficacy, and DNA damage induced by 222 nm far-UVC compared with conventional 254 nm UVC. (**a**) Emission spectrum of a Kr–Cl excimer lamp, showing a dominant peak at 222 nm with optical filtering to suppress longer-wavelength components. (**b**) Bacterial inactivation as a function of UV fluence, demonstrating comparable germicidal efficacy of 222 nm and 254 nm irradiation. (**c**) Induction of cyclobutane pyrimidine dimers (CPDs) in keratinocytes following UV exposure. (**d**) Induction of 6-4 photoproducts (6-4PPs) in keratinocytes as a function of UV fluence (Reprinted from ref. [14]).

The germicidal and inactivation nature of UVC stems from the strong absorption of short-wave ultraviolet radiation by nucleic acids. DNA and RNA exhibit an absorption peak around 260 nm; upon absorption, the most typical molecular event is the formation of photoproducts such as cyclobutene pyrimidine dimers (CPDs) between adjacent pyrimidine bases. This subsequently triggers replication and transcription blocks, leading to microbial inactivation [70]. Historically, clinical and public applications of UVC have been constrained by the conflict between “high-efficiency inactivation” and “human safety.” Traditional 254 nm light sources possess clear potential for damage to skin and ocular tissues and are therefore typically restricted to unoccupied spaces or strictly shielded conditions. Far-UVC (commonly 222 nm) proposes a critical biophysical window: due to the stronger absorption by protein/peptide bonds and other biological macromolecules at shorter wavelengths, the effective penetration depth of 222 nm in tissue is significantly reduced. This makes it difficult for radiation to reach the viable nucleated cell layers of human tissue, yet it remains capable of effectively inactivating micron/sub-micron scale targets such as bacteria and viruses. This provides a theoretical and empirical foundation for continuous disinfection in “human-occupied” scenarios [14,71]. As shown in Figure 13, filtered 222 nm Far-UVC forms a narrow-spectrum output. While achieving bacterial inactivation effects comparable to 254 nm UVC, it induces only extremely low levels of CPD and 6-4PP DNA damage, thereby supporting the safety window characteristic of “high-efficiency inactivation with low tissue penetration” at the physical mechanism level. Beyond in vitro, animal, and reconstructed skin models, more direct human validation has emerged recently. Sugihara et al. conducted safety experiments involving 222 nm intervention on the human eye, following up on ocular surface indicators under multi-dose exposure conditions. This work provides an evidence dimension closer to clinical reality for the risk assessment of Far-UVC in occupied spaces [72,73]. From the perspective of this review, the importance of these developments lies not only in wavelength-dependent germicidal efficacy but also in the growing need for dose-resolved safety characterization and deployment-aware quantitative control.

#### 5.1.2. Engineering and Applications

Beyond the “safety wavelength window,” the core bottleneck for the engineering implementation of UVC lies in the light source. Mercury lamps suffer from limitations such as bulkiness, fragility, and limited capabilities for modulation and array formation, making them ill-suited for the thin form factors and localized dosage control required by wearable or patch-type devices. Deep Ultraviolet (DUV) LEDs, represented by AlGaN materials, are viewed as the critical alternative route to mercury lamps. Their advantages lie in the potential for device miniaturization, array configuration, rapid modulation, and natural compatibility with portable equipment. However, the efficiency improvement of UVC-LEDs has long been constrained by issues such as defect density in high-Al content materials, carrier injection efficiency (particularly p-type), and light extraction efficiency. These factors have led to slow progress in improving external quantum efficiency (EQE) and output power [74,75]. In recent years, systematic improvements focusing on tunnel junction/injection layer design, defect and doping engineering, and light extraction structures are driving the evolution of UVC-LEDs toward higher efficiency and reliability. These advancements provide the device-level foundation for miniaturized UVC modules to enter fields such as medical sterilization, wound care, and embedded systems [76,77]. For translational deployment, especially in occupied or clinically adjacent settings, source engineering alone is not sufficient. Practical implementation also requires traceable irradiance characterization, control of spectral leakage, and quantitative verification of delivered dose under realistic operating conditions. In this sense, the translational value of UVC systems depends not only on source efficiency, but also on whether dose delivery can be measured and safety constraints can be enforced in a reproducible manner.

### 5.2. UVB (280–315 nm)

#### 5.2.1. Physical Mechanism

Unlike the direct inactivation mechanism of UVC, clinical UVB, particularly narrowband UVB (NB-UVB) centered at approximately 311–313 nm, primarily acts by reshaping the cutaneous immune microenvironment. In inflammatory skin diseases such as psoriasis, a key pathological feature is sustained activation of pathogenic T cells and the IL-23/IL-17 inflammatory axis. Studies on human lesions have shown that NB-UVB induces apoptosis of infiltrating T cells and downregulates IL-23/IL-17–related signaling, shifting plaques from a highly inflamed state toward transcriptional and immune profiles closer to normal skin [78,79]. From an engineering perspective, the value of NB-UVB is therefore not to maximize phototoxicity but to deliver a dose that is sufficient to achieve immune modulation while maintaining an acceptable risk of erythema. This balance makes UVB therapy intrinsically dose-sensitive and therefore closely linked to the need for reproducible delivery, target-site dose awareness, and patient-specific dose adjustment.

#### 5.2.2. Engineering and Applications

Traditional narrowband UVB (NB-UVB) therapy has long relied on large, hospital-based whole-body phototherapy cabins or handheld outpatient devices. While clinically effective, these systems have several practical limitations. They can impose a substantial travel and compliance burden on patients, and rigid light-delivery geometries often fail to account for body-surface curvature, local lesion distribution, shielding by adjacent tissue, or variations in source-to-skin distance. As a result, the nominal device setting may not accurately reflect the true dose delivered to the treatment site. Recent clinical evidence has strengthened the case for home-based NB-UVB. In particular, the LITE randomized clinical trial showed that home-based NB-UVB was non-inferior to office-based phototherapy for psoriasis in terms of efficacy and safety, while substantially reducing treatment burden [80]. However, the expansion of home phototherapy also makes dose supervision, adherence monitoring, and site-specific dose consistency more important, because the treatment environment is less controlled than in supervised clinical settings. In this context, wearable dosimetry, mobile reporting, and digitally assisted treatment logging may provide an important bridge between conventional open-loop phototherapy and more traceable dose-guided workflows.

Another important route is targeted phototherapy using 308 nm excimer light or lasers. By delivering energy through small, well-defined spots, this approach concentrates treatment on lesions, reduces irradiation of surrounding healthy skin, and can intensify dosing for recalcitrant plaques [81,82]. From a systems perspective, targeted UVB platforms also illustrate why sensing matters: the smaller and more localized the treatment field, the more sensitive treatment quality becomes to geometric alignment, lesion boundary definition, and reproducibility of delivered dose. Overall, current UVB therapy is still predominantly open loop in routine use, but it is increasingly compatible with sensing-assisted supervision, especially in home treatment, targeted delivery, and personalized dose escalation workflows.

### 5.3. UVA (315–400 nm)

#### 5.3.1. Physical Mechanism

Compared to UVC and UVB, the energy of a single UVA photon is significantly lower, making it difficult to directly induce photochemical DNA breakage or rapid cytotoxic reactions. Its biomedical effects are therefore primarily realized through indirect photochemical pathways mediated by photosensitizers. In this band, UVA acts more as an “energy trigger” rather than a direct molecular destructive factor.

**Figure 14:**
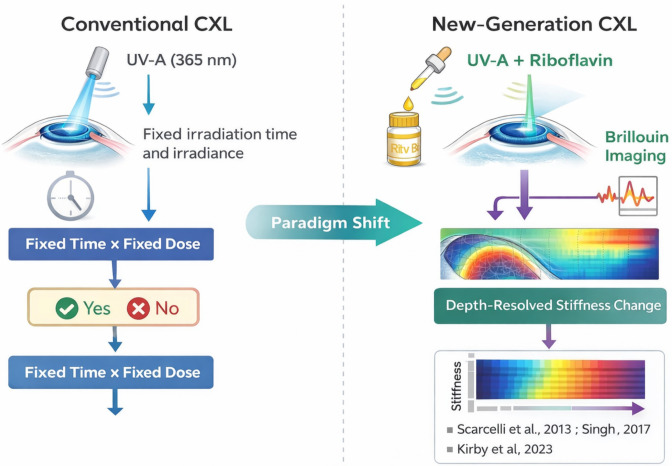
Paradigm shift in UVA corneal collagen crosslinking (CXL) from time–dose prescriptions to mechanics-based endpoints. Conventional CXL relies on fixed irradiation time and dose, yielding a binary treatment outcome, whereas next-generation CXL integrates UVA–riboflavin excitation with Brillouin imaging or optical coherence elastography to enable depth-resolved, quantitative assessment of corneal stiffening. Adapted from refs. [88,89,90]. The color scales in the depth-resolved stiffness profiles represent the mechanical gradient of the cornea, where cooler colors (blue) indicate lower stiffness and warmer colors (yellow to red/purple) indicate increased stiffness following crosslinking.

Corneal collagen crosslinking (CXL) is the most representative clinical example of this mechanism (as shown in Figure 14). After sufficient infiltration with riboflavin, UVA (typically 365 nm) excites the photosensitizer into a triplet state, which then transfers energy to surrounding oxygen molecules to generate reactive oxygen species (ROS) such as singlet oxygen. These highly reactive, short-lived intermediates promote the formation of new covalent crosslinks between collagen fibres, thereby significantly increasing the biomechanical strength of the corneal stroma and halting the progression of corneal ectasia without relying on extensive cellular damage [83].

The same UVA photochemistry framework also underlies key dermatologic UVA therapies. PUVA combines a psoralen photosensitizer with UVA exposure; psoralen photo adduct formation and related downstream effects support therapeutic benefit but also impose phototoxicity and long-term risk considerations [84,85]. In contrast, UVA1 (340–400 nm) emphasizes deeper dermal penetration and is used in several inflammatory dermatoses, with reported mechanisms including immunomodulatory effects (e.g., T-cell apoptosis and dermal cellular changes) and matrix-related pathways [86,87].

#### 5.3.2. Engineering and Applications

The engineering complexity of UVA-based therapy arises from the fact that incident light does not fully determine effective tissue response. In UVA-CXL, crosslinking efficacy and depth depend on the coupled effects of UVA photon flux in the stroma, oxygen diffusion and depletion kinetics, corneal thickness and heterogeneity, riboflavin distribution and optical screening, and endothelial safety thresholds. This coupling creates a narrow operating window: insufficient effective dose results in limited crosslinking depth and biomechanical gain, while excessive or poorly controlled delivery increases the risk of endothelial injury. These characteristics limit the generalizability of conventional open-loop prescriptions based on fixed irradiance and exposure time.

Accordingly, recent engineering trends have shifted from optimizing UVA sources alone toward integrating mechanical imaging as functional readouts to quantify treatment effect and support protocol optimization. Brillouin microscopy has been used to noncontact quantify depth-dependent biomechanical changes in corneas before and after UVA-CXL, providing an objective mechanical map that correlates with treatment conditions [88]. Optical coherence elastography (OCE) further enables spatially resolved evaluation of corneal elasticity changes and has been used to compare different crosslinking strategies, supporting individualized protocol design and safety window optimization [89,90].

Engineering considerations become even more explicit when UVA therapy is extended to dermatology. PUVA is intrinsically a multi-factor intervention: therapeutic outcome is jointly influenced by photosensitizer exposure and kinetics, the timing between drug administration and illumination, and the delivered UVA dose and geometry. This motivates systems that prioritize workflow consistency, dose traceability, and cumulative exposure management [84,85]. UVA1 treatments commonly require longer exposures and larger fields, shifting engineering constraints toward thermal load, irradiance uniformity over large areas, and session-to-session dose consistency; practical deployment therefore depends strongly on robust dosimetry and standardized delivery geometry [86,87].

### 5.4. AI-Enabled Precision Phototherapy

Clinical decisions in ultraviolet phototherapy are ultimately guided by tissue responses and clinically relevant outcomes, such as erythema risk, inflammation remission, and the extent of tissue remodelling, rather than irradiance or cumulative dose alone. Although flexible sensors can continuously measure skin-received dose, the dose–response relationship remains highly non-linear and varies across individuals and treatment contexts. It is influenced by pigmentation, lesion burden, tissue thickness, anatomical site, ambient light exposure, adherence behavior, and other sources of variability. For this reason, AI is best understood here not as an autonomous replacement for clinical judgment, but as a computational layer that may help connect measured dose, patient state, and predicted outcome within a more explicit decision framework.

At present, the most established AI-related contributions are found in patient-state standardization, image-based severity scoring, and multimodal risk stratification, rather than in fully validated real-time closed-loop UV dose control [91]. For example, deep learning has been used to quantify disease severity components relevant to psoriasis management and to improve the consistency of image-based phenotyping [92,93]. In parallel, broader work in medical AI has highlighted the importance of calibration, uncertainty estimation, and fairness across skin tones and imaging conditions when building individualized prediction models.

More advanced uses of AI in phototherapy, such as dynamic treatment adaptation, reinforcement-learning-based dose optimization, and integrated sensing-computing-treatment systems, should currently be viewed as early-stage or proof-of-concept rather than as routine clinical practice. Smart wound dressings and UVC-enabled therapeutic platforms illustrate the feasibility of combining sensing, control logic, and therapeutic actuation in localized settings [94,95,96], while reinforcement learning and dynamic treatment regime frameworks provide a conceptual basis for future dose adaptation under uncertainty and safety constraints [97]. However, evidence linking these computational strategies to durable clinical benefit in UV phototherapy remains limited, and external validation under realistic treatment conditions is still needed.

After treatment, AI may also support longitudinal efficacy assessment and non-invasive follow-up. Generative models have been applied to virtual staining and virtual pathology from label-free signals [88], and machine learning models have been explored for progression prediction in corneal collagen crosslinking [98,99]. Overall, AI currently contributes most credibly when it improves standardization, interpretation, or prediction around UV therapy, whereas fully closed-loop AI-driven phototherapy remains an emerging rather than established paradigm.

### 5.5. Challenges and Future

The efficacy and safety of UV therapy and disinfection depend on the spatial and temporal distribution of delivered dose, but this distribution is difficult to control under conditions of tissue heterogeneity, variable geometry, motion, and real-world workflow constraints. Narrow safety margins, particularly in UVC and Far-UVC applications, mean that current evidence is still insufficient to define long-term safety across all repeated low-dose human exposure scenarios. The recent review literature likewise emphasizes that, despite substantial progress, additional long-term and mechanism-aware safety studies remain necessary before far-UVC deployment can be considered fully resolved from a translational or regulatory standpoint [21]. In addition, many current therapeutic workflows remain predominantly open loop, with limited direct feedback on target-site dose or tissue response.

A central challenge for future development is therefore to distinguish clearly between three levels of maturity: conventional open-loop treatment, feedback-informed or partially monitored treatment, and genuinely closed-loop therapeutic control. At present, most clinically used UV systems fall into the first category, a smaller set of research platforms falls into the second, and only a limited number of experimental systems approach the third. This distinction is important for realistic interpretation of the field and for avoiding overstatement of current translational readiness.

Future work should emphasize real-time dose verification, better linkage between physical dose and biological response, uncertainty-aware treatment adjustment, and stronger clinical evidence using standardized endpoints and longer follow-up. For Far-UVC in particular, clearer guidance is needed regarding where deployment can be considered safe, measurable, and clinically justified. More broadly, progress in this area is likely to depend less on UV source power alone and more on whether sensing, dosimetry, and computational support can be integrated into treatment workflows in a traceable and clinically interpretable manner.

## 6. Challenges and Outlook

### 6.1. Challenges

Despite rapid progress across UV-enabled imaging, dosimetry, and therapy-related applications, the clinical translation of UV biomedical systems remains constrained by several unresolved cross-cutting challenges. These challenges do not arise from any single platform alone. Rather, they reflect a broader systems problem involving safety, quantitative reliability, standardization, workflow compatibility, and the interpretation of UV-derived signals under uncertainty. The first and most fundamental challenge is uncertainty in safety windows and real-world dose. The biological effects of UV exposure depend not only on wavelength and irradiance, but also on tissue type, cumulative exposure, spatial dose heterogeneity, and inter-individual variability [59,100]. Even in the case of Far-UVC at 222 nm, much of the available evidence still derives from short-term cell studies, reconstructed skin models, animal experiments, or relatively limited human observations [14,101]. Although these studies have substantially advanced the field, they do not yet define universally accepted long-term safety boundaries for repeated low-dose human exposure across diverse deployment scenarios. As a result, whether UV is used as an excitation source in imaging or as an irradiation source in disinfection and phototherapy, the absence of a sufficiently validated relationship between delivered dose, true tissue exposure, and biological effect remains a major barrier to auditability, clinical confidence, and safe large-scale deployment.

A second challenge is the lack of cross-platform standardization and quantitative consistency. For UV-PAM and MUSE, many reports remain optimized under platform-specific conditions involving different wavelengths, pulse parameters, optical geometries, specimen handling procedures, staining workflows, and post-processing pipelines [12,13,102]. This makes comparison across studies difficult and reduces confidence in reproducibility. In this context, standardization should be understood not merely as a matter of reporting style but as an enabling condition for translation. Shared definitions of image quality, uncertainty, calibration procedure, specimen preparation, and task-based performance remain limited across the field [22]. Reviews of photoacoustic imaging have already emphasized that without standardized calibration phantoms, unified evaluation frameworks, and physically constrained correction workflows, multi-center validation cannot produce a reproducible and auditable chain of evidence [20]. Establishing traceable specifications for spectral calibration, angular-response correction, and compensation for environmental drift is therefore necessary for dose evidence to meet regulatory expectations. Similar issues arise in wearable UV dosimetry, where differences in spectral response, angular response, integration time, skin conformity, and environmental drift complicate comparison across devices and studies [18,19,23]. Progress toward clinical or public-health deployment will therefore require more traceable calibration procedures, explicit uncertainty reporting, and common evaluation conventions that are usable across laboratories, devices, and use scenarios.

A third challenge concerns the persistent gap between nominal exposure and biologically relevant dose. This gap appears in different forms across the domains reviewed here. In UV-PAM, measured signal is shaped by fluence distribution, optical attenuation, acoustic coupling, and transducer response. In MUSE, final image quality depends not only on UV excitation but also on staining consistency, surface flatness, optical focus, and computational processing. In wearable UV dosimetry, measured irradiance or cumulative exposure can differ substantially from true skin-received or biologically effective dose because of posture, curvature, placement, occlusion, sweating, sunscreen use, and skin-specific factors. In UV therapy, prescribed settings often do not correspond directly to the dose delivered to the treatment site or to the resulting biological response. Taken together, these examples indicate that the central translational problem across UV biomedical systems is not signal acquisition alone, but signal interpretation under incomplete observability. A clinically useful system must therefore specify what quantity is being measured, how it was calibrated, and with what degree of uncertainty it can support a downstream decision.

A fourth challenge is the generalization, interpretability, and safe use of artificial intelligence. Deep learning has clearly improved virtual staining, reconstruction, domain correction, and automated interpretation in UV-enabled imaging [10,51], and it is increasingly being explored for dose interpretation and treatment-related decision support. However, AI performance in these settings remains sensitive to training distribution, device configuration, sample preparation, wavelength regime, and population heterogeneity. In less standardized modalities such as UV imaging, these issues are amplified because signal characteristics can shift substantially with acquisition conditions and tissue handling. Moreover, when AI is used to generate pathology-like images or guide therapeutic decisions, hallucinated features, miscalibrated confidence, or biased predictions become clinically consequential. For this reason, future UV-AI systems should not be assessed only by average performance metrics. They should also report robustness under domain shift, calibration quality, uncertainty estimates, preservation of decision-relevant features, and results from external validation.

A fifth challenge, which remains underdeveloped in much of the current literature, is regulatory and ethical readiness. Moving from laboratory prototypes to clinical or quasi-clinical deployment requires engagement with device classification, quality assurance, calibration traceability, human factors, and the distinction between research-use and clinical-use systems. In practical terms, this means that UV-based biomedical platforms should be considered in relation to existing medical-device and software-related regulatory frameworks, including FDA guidance for software as a medical device and AI/ML-enabled medical devices, as well as European CE-marking pathways under the Medical Device Regulation or In Vitro Diagnostic Regulation where applicable [103,104]. For systems that generate pathology-like images or support treatment decisions, regulatory assessment should also consider analytical validity, clinical validity, usability, risk management, and post-deployment monitoring. This is particularly important for platforms that generate pathology-like outputs, influence treatment dosing, or are deployed in occupied environments. For Far-UVC systems, long-term human safety uncertainty remains directly relevant to regulatory review and deployment decisions. For AI-assisted platforms, ethical concerns include interpretability, accountability, bias across skin tones and patient groups, and the risk of over-reliance on outputs that appear clinically intuitive but are not yet fully trustworthy. Accordingly, translational progress in this field will depend not only on technical performance, but also on whether UV systems can be made measurable, explainable, quality-assured, and governable within real biomedical settings.

Taken together, these challenges suggest that the future of UV biomedical systems depends less on maximizing isolated technical metrics and more on strengthening the full chain from physical signal generation to calibrated readout, uncertainty-aware interpretation, and clinically responsible use.

### 6.2. Outlook

Looking ahead, UV biomedical engineering is moving from isolated performance optimization toward system-level integration. The next stage of progress is likely to depend on the convergence of four elements: standardized physical measurement, platform-level calibration, AI-assisted interpretation, and decision-oriented workflow design. In imaging, this means that future UV-PAM and MUSE systems will need to be judged not only by resolution, contrast, or throughput, but also by reproducibility, uncertainty reporting, and pathology task performance under realistic sample and workflow conditions. In dosimetry, the focus is likely to shift from recording exposure alone toward estimating tissue-relevant dose and linking that estimate to individualized guidance or treatment supervision. In therapy, future progress will depend on whether dose delivery can be made more traceable and whether sensing can be integrated into adaptation strategies without overstating current closed-loop maturity.

At the hardware and platform level, continued advances in DUV LEDs, photodetectors, acoustic detection hardware, compact optics, and flexible electronics are expected to improve integration, reproducibility, and cost-performance balance [7,8,9]. However, hardware progress alone will not be sufficient. A shared translational infrastructure is also needed, including standardized calibration protocols, benchmark datasets, common reporting conventions, and evaluation criteria that extend beyond proof-of-concept demonstration. Such infrastructure will be essential if UV technologies are to progress from isolated laboratory platforms to multi-center studies, regulatory assessment, and clinically credible deployment.

Future AI development in this field is also likely to become more structured and more constrained. Rather than relying solely on end-to-end data-driven models, the next phase will likely emphasize physics-informed and calibration-aware AI that incorporates optical, acoustic, dosimetric, and anatomical priors into model design. This is especially relevant in UV systems, where measured signals are often shallow, heterogeneous, and tightly coupled to acquisition conditions. In imaging, future AI may support uncertainty-aware virtual histology, cross-platform domain adaptation, and multimodal fusion with OCT, white-light imaging, pathology reference data, or clinical metadata. In dosimetry, AI is likely to become increasingly important for geometric correction, individualized risk estimation, and the translation of imperfect sensor measurements into biologically meaningful dose estimates. In therapy, promising directions include patient-specific predictive models, state-aware treatment optimization, and data-guided dose adjustment under explicit safety constraints. Recent work also suggests that UV-based label-free sensing can be extended beyond imaging by coupling absorbance spectroscopy with machine learning for rapid biomedical screening and decision support, indicating a broader future role for AI-assisted UV readout in translational workflows [105]. Even so, these future AI-enabled systems should be viewed as emerging opportunities rather than imminent replacements for clinical judgment. Their value will depend on external validation, interpretability, fairness, and calibration-aware deployment.

Another important future direction is the use of more explicit maturity models for feedback-enabled UV systems. At present, the language of intelligent control or closed-loop adaptation is sometimes applied even when actual implementations remain open-loop or only partially monitored. A more precise framework would distinguish among three levels: conventional open-loop systems, feedback-informed systems with measurable but limited adaptation, and genuinely closed-loop systems in which sensing, state estimation, and intervention are tightly integrated. Adopting such a distinction would improve both scientific clarity and translational realism, especially in therapy-related applications.

From a clinical perspective, UV technologies are more likely to complement than replace established methods. MUSE is likely to remain most valuable as a rapid slide-free assessment tool for intraoperative decision support and low-resource pathology rather than as a full substitute for routine H&E [13,102]. UV-PAM is particularly promising for margin assessment, label-free histology-like readout, and settings where endogenous nuclear contrast provides a practical advantage [12,22]. Wearable UV dosimetry may become increasingly important as the quantitative interface between environmental exposure, home phototherapy, and individualized prevention. In disinfection and phototherapy, the most useful future systems are likely to be those that combine delivery with measurable safety, dose traceability, and interpretable feedback rather than relying on nominal source settings alone.

Overall, the next stage of progress will depend on whether UV technologies can be developed as measurable, standardized, and decision-guiding biomedical systems rather than as isolated optical tools. In practical terms, this will require coordinated advances in safety modeling, standardization, calibration, ethically responsible AI, and clinically meaningful workflow integration.

## 7. Conclusions

In summary, ultraviolet technologies are becoming increasingly valuable not simply as irradiation tools but as sensing-guided biomedical systems that support quantitative readout, dose monitoring, and feedback-informed intervention. Across ultraviolet photoacoustic microscopy (UV-PAM), microscopy with ultraviolet surface excitation (MUSE), wearable and embedded UV dosimetry, and sensor-assisted phototherapy, a shared engineering logic can be identified: biologically meaningful contrast or response must first be transduced into measurable signals, then calibrated, interpreted, and linked to downstream decisions. From this perspective, the central contribution of UV is not only wavelength-specific biointeraction but also its ability to generate surface-sensitive, label-free, and dose-relevant information for diagnosis support and treatment guidance.

At the same time, the clinical and translational value of UV systems still depends on whether sensing outputs can be made reliable, comparable, and actionable across devices and use scenarios. Key barriers remain in safety-window uncertainty, true skin-dose estimation, cross-platform calibration, and the robustness and interpretability of AI-assisted analysis. Addressing these issues will require stronger standardization of readout metrics, traceable dosimetric workflows, physically informed correction models, and multimodal validation under realistic operating conditions. Overall, the field is moving from largely open-loop UV use toward more feedback-informed systems in which sensing, computation, and intervention are increasingly integrated, although fully closed-loop operation remains limited in current practice. Framing UV platforms through the common language of signal acquisition, quantitative readout, uncertainty control, and feedback design helps clarify their relevance to biosensors-oriented biomedical engineering. Future progress will therefore depend less on increasing optical power alone, and more on building UV systems that are measurable, auditable, and decision-guiding in real biomedical environments.

## Figures and Tables

**Table 1:** Comparison of the present review with representative prior reviews related to UV-enabled biomedical imaging, dosimetry, and therapy.

Reference	Year	Primary Domain	Main Scope	Imaging	Dosimetry	Therapy/Disinfection	AI	Calibration	Translational	Key Limitation Relative to the Present Review
[17]	2019	Ex vivo microscopy/pathology	Reviews ex vivo microscopy platforms for surgical pathology practice	Partial	No	No	Limited	Limited	Major	Focused on pathology microscopy workflows rather than UV-centered biomedical systems; does not integrate dosimetry or therapeutic guidance.
[18]	2021	Wearable/portable UV sensing	Review of wearable and portable sensors for personal solar UV exposure monitoring	No	Major	Limited	Limited	Partial	Partial	Concentrates on exposure monitoring and sensor form factors; does not connect dosimetry with UV imaging or therapy in a unified framework.
[19]	2022	Wearable UV dosimetry	Review of wearable UV radiation sensors for research and personal use	No	Major	Limited	Limited	Partial	Partial	Strong on device comparison but centered on personal UV monitoring rather than cross-domain biomedical system integration.
[20]	2024	Quantitative photoacoustic imaging	Reviews challenges, calibration, and solutions in quantitative photoacoustic imaging	Partial	No	No	Limited	Major emphasis	Partial	Emphasizes quantification and calibration in photoacoustic but is not UV-specific and does not extend to dosimetry or therapy.
[21]	2024	Far-UVC safety/disinfection	Reviews safety considerations for new germicidal Far-UVC technologies	No	Limited	Major	No	Partial	Partial	Strong on safety and deployment questions but not organized around biosensing, imaging, or dosimetric system integration.
[11]	2025	Ultraviolet microscopy	Reviews recent advances in ultraviolet microscopy across instrumentation and applications	Major	No	No	Partial	Limited	Partial	Strong on UV microscopy but does not integrate wearable dosimetry, therapeutic workflows, or cross-platform calibration logic.
[22]	2025	Photoacoustic microscopy	Comprehensive review of high-performance PAM systems	Major emphasis	No	No	Partial	Partial	Partial	Focused on PAM system performance and design rather than UV-enabled biomedical systems spanning imaging, dosimetry, and therapy.
[23]	2025	UV thin-film dosimetry	Reviews properties and applications of ultraviolet radiation thin-film dosimetry	No	Major	Limited	No	Partial	Partial	Strong on thin-film dosimetry materials and applications but does not connect dosimetry to imaging, AI, or therapeutic feedback workflows.
This review	2026	Cross-domain UV biomedical systems	Integrates UV-PAM, MUSE, wearable/embedded UV dosimetry, and sensing-assisted UV therapy under a common biosensing-informed framework	Major	Major	Major	Major	Major	Major	Provides a cross-domain synthesis organized by physical mechanism, transduction, quantitative readout, calibration, AI-assisted analysis, and translational constraints.

**Table 4:** System configurations, workflow characteristics, and clinical translation of microscopy with ultraviolet surface excitation (MUSE).

Ref.	Year	UV Excitation (nm)	Excitation Source	Optical Configuration	Staining Time	Effective Imaging Depth	Field of View	Time to Image	Reconstruction/Processing	Clinical Use Case	Key Workflow Advantage
[13]	2017	<300	UV lamp/LED	Dry, wide-field	~10–30 s	~10–20 µm	mm^2^-scale	<1 min	Pseudo-coloring	Skin, breast	First slide-free UV surface histology
[42]	2018	~280	UV LED	Dry	~1 min	~10 µm	mm^2^-scale	Minutes	Simple color mapping	Dermatopathology	Rapid Mohs-style assessment
[43]	2018	~280	UV LED	Immersion	<1 min	~10–20 µm	cm^2^-scale	Few minutes	Mosaic stitching	Breast margins	Large-area margin screening
[45]	2019	~280	UV LED	Immersion	<1 min	~10–20 µm	cm^2^-scale	Minutes	Automated mosaicking	Breast surgery	Whole-margin coverage
[47]	2021	<300	UV LED	Compact	Seconds	~10 µm	mm^2^-scale	<1 min	On-device processing	POC/field use	Democratized pathology
[51]	2021	~280	UV LED	Immersion	<1 min	~10–20 µm	mm^2^-cm^2^	Minutes	DL virtual staining	General pathology	Reduced interpretation barrier
[48]	2024	<300	Fiber-coupled UV	Fiber-based	Seconds	Surface-limited	mm^2^-scale	Near real-time	Generative restoration	Endoscopic imaging	In vivo extension
[50]	2022	<300	UV LED	Serial surface imaging	Seconds/slice	Surface-limited	Organ-scale (3D)	Minutes–hours	Volumetric reconstruction	Whole-organ atlas	Rapid 3D histology

Note: The workflow characteristics summarized here are reported under study-specific implementation conditions. Differences in specimen type, staining workflow, optical configuration, and reconstruction pipeline should be considered when interpreting these values across systems.

**Table 5:** Quantitative evaluation of AI-based methods in MUSE microscopy.

Functional Category	Ref.	Methodology/Model	Performance Metric	Quantitative Value	Compared to	Key Technical Merit
Virtual staining	[51]	Deep-MUSE (dual-path GAN)	SSIM	0.37 ± 0.08	Pix2Pix baseline	Automated virtual H&E generation from UV-excited fluorescence with preserved structural consistency.
			PSNR	19.5 ± 1.1 dB		Improved perceptual fidelity under slide-free, unregistered imaging conditions.
			Pearson correlation	0.73 ± 0.05		Higher cross-modality correlation between virtual and reference H&E images on validation data.
	[52]	CycleGAN	Critic accuracy	71.9%	Pix2Pix/color-mapping baseline	Effective unpaired modality translation without pixel-aligned ground truth.
	[53]	Spectral Pix2Pix + PCA (5 components)	FID	0.0614	Full-spectrum input	Improved generative realism with >80% spectral dimensionality reduction.
			SSIM	0.686		Preserved structural similarity despite aggressive spectral compression.
Image restoration	[54]	DL-EDOF	PSNR gain	+3–6 dB	Conventional EDOF methods	Robust blind deblurring and depth-of-field extension without mechanical scanning.
	[48]	FUSE-Flow (normalizing flow)	Uncertainty threshold	σ ≥ 5 (*p* < 3 × 10^−7^)	GAN-based enhancement	Explicit pixel-wise uncertainty quantification to mitigate hallucination risk.
System synergy	[55]	MALS + reconstruction	DOF extension factor	16×–35×	Standard DOF imaging	Ultra-large depth-of-field extension via hardware–software co-design.

Note: The reported AI-related metrics are task-specific and dataset-dependent. Because studies differ in imaging modality, dataset composition, preprocessing, ground-truth definition, and evaluation protocol, these values should not be interpreted as directly comparable indicators of overall model superiority across methods.

**Table 6:** Wearable and embedded UV dosimetry: comparison of environmental exposure versus true skin-level dose.

Ref.	Sensing Principle	Power	Spectral Selectivity	Angular Correction	Skin Conformality	Output	Clinical/Application Scenario	Key Limitation (Truthfulness/Fidelity)
[59]	Measurement framework (radiometry)	—	—	—	—	—	Metrology foundation	Not a wearable system; enables definitions/traceability only
[23]	Colorimetric/thin-film dosimetry (review)	Passive	Often UVB-weighted (reviewed)	None/limited	Flat/film	Cumulative	Environmental exposure/research badges	Strong angle dependence; usually ambient-oriented; limited real-time capability
[19]	Wearable UV sensors (review)	Battery/passive (varied)	UVA/UVB (varied)	Often none	Wrist/clip/patch (varied)	Real-time/cumulative	Personal monitoring/research & consumer devices	Highlights heterogeneity; many devices measure ambient UV; orientation/placement major error source
[18]	Wearable/portable personal solar UV exposure sensors (review)	Battery/passive (varied)	UVA/UVB (varied)	Identifies cosine error; discusses mitigation strategies	Varied	Varied	Personal solar exposure metrology	Orientation/behavior/environment confounding; difficult to infer true skin-dose without correction
[56]	Colorimetric epidermal patch + NFC readout	Passive sensing + NFC readout	UVA/UVB (as described in your Figure 9 context)	None (primarily)	Epidermal (skin-mounted)	Cumulative	Individual exposure logging; population monitoring	Still sensitive to incident geometry; cumulative only; calibration needed
[58]	Epidermal electronics mechanics paradigm	—	—	Mechanical mitigation (contact stability)	Epidermal	Enables stable measurement	Mechanical prerequisite for skin-dose fidelity	Not a UV sensor by itself; must integrate sensing + calibration
[9]	Flexible miniaturized dosimeter system (wireless, battery-free)	Battery-free/wireless	Applied to solar + phototherapy contexts	In situ placement reduces geometry gap	Flexible/skin-proximal	Real-time + cumulative	Phototherapy supervision + calibration; exposure monitoring	Placement-dependent; needs QA/standardization; still not full-body dose field
[57]	Photoelectronic UV photodetector (device-level)	Self-powered detection (device)	UV-selective via wide-bandgap design	None (device level)	Flexible (device-level)	Real-time irradiance (device)	Enabling component for wearables	Device output ≠ true skin-dose unless geometry/contact/algorithm handled
[60]	Wearable sensor + mobile feedback (behavior loop)	Battery (typical wearable)	—	Not explicit	Wearable	Real-time feedback	Sun-exposure reduction interventions	Effectiveness depends on measurement fidelity; risk of bias if orientation error not corrected
[61]	Wearable UV dosimeter (trial)	—	—	Not explicit	Wearable	Real-time/cumulative	RCT evidence for prevention	Clinical impact depends on adherence + accuracy; many devices not skin-dose calibrated

Note: The approaches summarized here are reported under different sensing principles, calibration strategies, body placements, and use scenarios. Device-level output and true skin-level dose fidelity are not equivalent, and cross-study interpretation should therefore consider geometry, spectral response, and application context.

**Table 7:** One table view of UV-enabled platforms across imaging, dosimetry, and treatment (UV-PAM, MUSE, wearable/embedded dosimetry, and phototherapy).

Platform	Target	Transduction	Calibration	Main Uncertainty Sources	Decision Output
UV-PAM	DNA/RNA absorption and nuclear features in tissue	UV light makes a photoacoustic signal, an ultrasonic transducer detects it (light → sound)	Set UV wavelength and UV dose; keep fluence even; set scan geometry; keep acoustic contact stable; keep transducer response stable	Uneven fluence; light loss in tissue; weak or unstable acoustic contact; transducer response limits; motion and scan artifacts; tissue differences	Fast histology-like nuclear readout; margin check; AI-based virtual staining, image recovery, and diagnosis support
MUSE	Tissue surface features, with fast staining in many cases	UV excites the surface, tissue gives visible fluorescence, a camera reads it (UV → visible)	Control stain time and method; keep surface contact stable; set optics and focus; keep color mapping stable	Uneven surface and focus; uneven staining; bleaching; stitching errors; device-to-device shift; tissue condition changes	Fast slide-free histology-like images; intraoperative and point-of-care screening; AI-based virtual staining and image reading
Wearable UV dosimetry	True skin-level UV dose	Color change films or UV detectors; output is dose or dose rate	Match sensor spectrum to UV source; correct angle error; keep good skin contact; map device reading to true skin dose	Angle error; spectrum mismatch; poor skin contact and curvature; motion and placement changes; sweat/skin effects; drift and unit-to-unit differences	Personal exposure record; therapy dose verification; supports feedback-informed dose supervision and provides a basis for future closed-loop control
UV phototherapy	Treatment effect and safety (clinical outcome, risk of skin damage), not dose alone	UV light is the treatment input; feedback uses dose data (dosimeters) plus clinical or imaging readouts; AI links dose and state to outcome	Make dose traceable at the target site; set safety limits; link dose to outcome with simple models; estimate uncertainty for safe updates	Strong person-to-person differences; non-linear dose–response; uneven light on skin; limits in long-term safety data; no single clean model from true dose to effect	Personalized dosing support; safer dose adjustment under explicit constraints; feedback-informed treatment planning; supports smart dressing and sensing-assisted treatment systems

Note: This table is intended as a conceptual cross-platform synthesis rather than a normalized technical comparison. The listed calibration needs, uncertainty sources, and decision outputs are meant to summarize representative engineering considerations across platforms.

## Data Availability

No new data were created or analyzed in this study.

## Abbreviations

The following abbreviations are used in this manuscript:

AI	artificial intelligence
AI-MUSE	AI-assisted Microscopy with Ultraviolet Surface Excitation
AUC	area under the receiver operating characteristic curve
BF-H	brightfield hematoxylin and eosin (H&E)
BK7	borosilicate crown optical glass
CAD	computer-aided diagnosis
CMOS	complementary metal–oxide–semiconductor
CNN	convolutional neural network
CNR	contrast-to-noise ratio
CPD	cyclobutane pyrimidine dimer
CUBIC	Clear, Unobstructed Brain/Body Imaging Cocktails and Computational analysis
CXL	corneal collagen crosslinking
DL	deep learning
DL-EDOF	deep-learning-based extended depth of field
DNA	deoxyribonucleic acid
DOF	depth of field
DUV	deep ultraviolet
EDOF	extended depth of field
EQE	external quantum efficiency
FAD	flavin adenine dinucleotide
FID	Fréchet inception distance
FOV	field of view
FUSE	model name used in cited work (FUSE-Flow)
FWHM	full width at half maximum
GAN	generative adversarial network
GM-UV-PAM	galvanometer mirror-based ultraviolet photoacoustic microscopy
IL-17	interleukin-17
IL-23	interleukin-23
LC	liquid crystal
LED	light-emitting diode
LITE	Light Treatment Effectiveness (LITE) study
MALS	micro-mirror array lens system
MEMS	micro-electro-mechanical systems
MUSE	Microscopy with Ultraviolet Surface Excitation
NA	numerical aperture
NADH	nicotinamide adenine dinucleotide (reduced form)
NB-UVB	narrowband UVB
NFC	near-field communication
OCE	optical coherence elastography
OCT	optical coherence tomography
OR	optical resolution
OR-PAM	optical-resolution photoacoustic microscopy
PAM	photoacoustic microscopy
PASI	Psoriasis Area and Severity Index
PCA	principal component analysis
POC	point-of-care
PRR	pulse repetition rate
PSNR	peak signal-to-noise ratio
PUVA	psoralen plus UVA
QA	quality assurance
R0	complete tumor resection with negative margins (R0 resection)
RCT	randomized controlled trial
RNA	ribonucleic acid
ROC	receiver operating characteristic
ROI	region of interest
ROS	reactive oxygen species
SNR	signal-to-noise ratio
SSIM	structural similarity index measure
STAR	A*STAR (Agency for Science, Technology and Research)
TRUST	translational rapid ultraviolet-excited sectioning tomography
UV	ultraviolet
UV-A	ultraviolet A (UVA)
UV-AI	ultraviolet and artificial intelligence
UV-B	ultraviolet B (UVB)
UV-PAM	ultraviolet photoacoustic microscopy
UV-TUT	UV-transparent ultrasound transducer
UVA	ultraviolet A
UVA-CXL	UVA corneal collagen crosslinking
UVA1	long-wave UVA (340–400 nm)
UVB	ultraviolet B
UVC	ultraviolet C
UVC-LED	UVC light-emitting diode
UVGI	ultraviolet germicidal irradiation
